# Long noncoding RNA UPK1A-AS1 indicates poor prognosis of hepatocellular carcinoma and promotes cell proliferation through interaction with EZH2

**DOI:** 10.1186/s13046-020-01748-y

**Published:** 2020-10-29

**Authors:** Dong-Yan Zhang, Qing-Can Sun, Xue-Jing Zou, Yang Song, Wen-Wen Li, Ze-Qin Guo, Shan-Shan Liu, Li Liu, De-Hua Wu

**Affiliations:** 1 Department of Radiation Oncology, Nanfang Hospital, Southern Medical University, Guangzhou, 510515 Guangdong Province China; 2 Hepatology Unit and Department of Infectious Diseases, Nanfang Hospital, Southern Medical University, Guangzhou, 510515 Guangdong Province China

**Keywords:** UPK1A antisense RNA 1, EZH2, Long non-coding RNA, miR-138-5p, Proliferation, Hepatocellular carcinoma

## Abstract

**Background:**

Dysregulation of long non-coding RNAs (lncRNAs) is responsible for cancer initiation and development, positioning lncRNAs as not only biomarkers but also promising therapeutic targets for cancer treatment. A growing number of lncRNAs have been reported in hepatocellular carcinoma (HCC), but their functional and mechanistic roles remain unclear.

**Methods:**

Gene Set Enrichment Analysis was used to investigate the molecular mechanism of UPK1A antisense RNA 1 (UPK1A-AS1). Cell Counting Kit-8 assays, EdU assays, flow cytometry, western blotting, and xenograft assays were used to confirm the role of UPK1A-AS1 in the proliferation of HCC cells in vitro and in vivo. Bioinformatics analyses and quantitative polymerase chain reaction (qRT-PCR) were performed to explore the interplay between UPK1A-AS1 and enhancer of zeste homologue 2 (EZH2). RNA immunoprecipitation (RIP), RNA pull-down assays, western blotting, and qRT-PCR were conducted to confirm the interaction between UPK1A-AS1 and EZH2. The interaction between UPK1A-AS1 and miR-138-5p was examined by luciferase reporter and RIP assays. Finally, the expression level and prognosis value of UPK1A-AS1 in HCC were analyzed using RNA sequencing data from The Cancer Genome Atlas datasets.

**Results:**

We showed that UPK1A-AS1, a newly identified lncRNA, promoted cellular proliferation and tumor growth by accelerating cell cycle progression. Cell cycle-related genes, including CCND1, CDK2, CDK4, CCNB1, and CCNB2, were significantly upregulated in HCC cells overexpressing UPK1A-AS1. Furthermore, overexpression of UPK1A-AS1 could protect HCC cells from cis-platinum toxicity. Mechanistically, UPK1A-AS1 interacted with EZH2 to mediate its nuclear translocation and reinforce its binding to SUZ12, leading to increased H27K3 trimethylation. Targeting EZH2 with specific small interfering RNA impaired the UPK1A-AS1-mediated upregulation of proliferation and cell cycle progression-related genes. Moreover, miR-138-5p was identified as a direct target of UPK1A-AS1. Additionally, UPK1A-AS1 was significantly upregulated in HCC, and the upregulation of UPK1A-AS1 predicted poor prognosis for patients with HCC.

**Conclusions:**

Our study revealed that UPK1A-AS1 promotes HCC development by accelerating cell cycle progression through interaction with EZH2 and sponging of miR-138-5p, suggesting that UPK1A-AS1 possesses substantial potential as a novel biomarker for HCC prognosis and therapy.

**Supplementary Information:**

The online version contains supplementary material available at 10.1186/s13046-020-01748-y.

## Background

Hepatocellular carcinoma (HCC) is the sixth most common malignancy and the fourth leading cause of cancer-related mortality worldwide [1]. Surgical resection, radiofrequency ablation, liver transplantation, chemotherapy, molecular-targeted therapy, and immunotherapy have been applied in HCC treatment [2, 3]. However, the survival rate remains unsatisfactory in patients with HCC because typical clinical features and specific indicators in the early stage of HCC are lacking [4]. Therefore, there is an urgent need to discover and develop more effective biomarkers and targets for better diagnosis, prognosis, and treatment of HCC.

With thousands of non-coding RNAs being identified and annotated, researchers have come to realize the great importance of non-coding RNA, which makes up more than 90% of the human genome [5]. Non-coding RNAs can be classified as microRNAs and long non-coding RNAs (lncRNAs) according to their size [6]. LncRNAs are transcripts of more than 200 nucleotides (nt) in length and possess no or limited protein-coding potential [7]. Recently, the importance of lncRNAs in tumorigenesis has gradually come to light [8]. LncRNAs have been recognized as key regulators involved in many biological processes rather than byproducts of RNA polymerase II transcription or genomic noise [9]. Increasing evidence has demonstrated that lncRNAs participate in regulating carcinogenesis through multiple pathways, including transcription modulation, post-transcription modulation, epigenetic modification, and RNA decay [10]. LncRNAs have also been regarded as potential biomarkers and therapeutic targets for cancers, including HCC [11]. For instance, lncRNA MCM3AP-AS1, an oncogenic lncRNA that is highly expressed in HCC, promotes the growth of HCC by targeting the miR-194-5p/FOXA1 axis [12]. LncRNA TUG1 is overexpressed in HCC and promotes proliferation by epigenetically silencing KLF2 [13]. DILC represses the self-renewal of cancer stem cells by inhibiting the autocrine IL-6/STAT3 axis [14]. In contrast, our previous findings showed that MIR22HG, a highly conserved lncRNA, was downregulated and predicted poor prognosis in patients with HCC [15]. These findings indicate that lncRNAs are critically involved in the development and progression of HCC and may serve as biomarkers for HCC diagnosis and prognosis.

UPK1A antisense RNA 1 (UPK1A-AS1) is a newly discovered lncRNA with little information about its functional role and clinical significance in cancers. It has been reported that UPK1A-AS1 is downregulated in esophageal squamous cell carcinoma (ESCC), and suppresses proliferation, migration, and invasion of ESCC cells by sponging microRNA-1248 [16]. To date, no study has reported the biological role and clinical importance of UPK1A-AS1 in HCC. Here, we determined the functional role of UPK1A-AS1 in HCC progression and uncovered the underlying molecular mechanism. Our results showed that UPK1A-AS1 was overexpressed in HCC, and the upregulation of UPK1A-AS1 predicted poor prognosis in HCC patients. Functionally, UPK1A-AS1 promoted proliferation by accelerating the G1/S transition of HCC cells. UPK1A-AS1 exerted its oncogenic activity by binding with EZH2 to mediate its nuclear translocation and reinforce its binding to SUZ12. Additionally, UPK1A-AS1 promoted HCC cell proliferation in part by sponging miR-138-5p. Our results uncovered the critical role of UPK1A-AS1 in HCC progression, and UPK1A-AS1 might serve as a potential biomarker for HCC diagnosis and prognosis.

## Methods

### Cell lines and cell culture

The human hepatocyte cell line L02 and HCC cell lines MHCC-97H, SK-Hep-1 Huh7, HCC-LM3, and Hep3B were provided by the Cell Bank of Type Culture Collection (CBTCC, Chinese Academy of Science, Shanghai, China). Cells were maintained in Dulbecco’s modified Eagle medium with 10% fetal bovine serum (Gibco, USA) and cultured in a humidified incubator containing 5% CO_2_ at 37 °C.

### Small Interference RNAs (siRNA) and lentivirus transduction

The siRNAs used in the current study were designed and synthesized by Ribobio Technology (Guangzhou, China). The transfection of siRNAs was performed according to the manufacturer’s instructions using Lipofectamine RNAiMAX Reagent (Invitrogen, USA). For lentivirus UPK1A-AS1 overexpression and knockdown, full-length UPK1A-AS1 was inserted into GV438, and two hairpin precursors specifically targeting UPK1A-AS1 were cloned into GV112. SK-Hep-1 and MHCC-97H cells were infected with lentivirus at a multiplicity of infection of 20 using polybrene (GeneChem, Shanghai, China). The sequences of siRNAs and target sequences of short hairpin RNAs (shRNAs) are listed in Supplementary Table 1.

### Gene Set Enrichment Analysis (GSEA)

GSEA was carried out using the GSEA program provided by Broad Institute (http://www.broadinstitute.org/gsea/index.jsp) to examine the gene sets or signatures associated with UPK1A-AS1or EZH2 in HCC samples from The Cancer Genome Atlas (TCGA) dataset. RNA sequencing (RNA-seq) data of HCC samples were downloaded from TCGA project, followed by GSEA analysis. An ordered list of all genes was generated according to their correlation with UPK1A-AS1 or EZH2, and predefined gene sets or signatures received an enrichment score and *P*-value.

### RNA extraction and Quantitative Polymerase Chain Reaction (qRT-PCR)

Total RNA was isolated from HCC cells and tumor tissues using TRIzol reagent (Invitrogen, USA) following the manufacturer’s instructions, and a total of 500 ng RNA was reverse transcribed into cDNA using a cDNA Reverse Transcription Kit (Takara, Japan). Quantitative PCR was performed using the SYBR Green PCR kit (Takara, Japan). β-actin was used as an internal control. Primer sequences used in the present study are shown in Supplementary Table 1.

### Cell proliferation assay

The cell proliferation assay was performed using the Cell Counting Kit (CCK)-8 Kit and Cell-Light EdU Apollo 567 in Vitro Imaging Kit (Ribobio Technology, Guangzhou, China). A total of 2000 cells were seeded in 96-well plates with indication treatments, and the CCK-8 proliferation assay was performed according to the manufacturer’s instructions. The EdU dye assay was performed according to the manufacturer’s recommendations.

### Cell cycle analysis and apoptosis assay

Cell cycle distribution was tested by flow cytometry on a FACScan (Beckman Instruments, USA). Cell apoptosis was detected using an Annexin V-FITC kit (Keygen Biotech, China).

### Western blotting assay

Proteins were separated on a sodium dodecyl sulfate-polyacrylamide gel (SDS-PAGE) and transferred onto polyvinylidene fluoride membranes (Bio-Rad, USA). The membranes were blocked with 5% bovine serum albumin (BSA) for 50 min at room temperature before incubation with primary antibody at 4 °C overnight. The membranes were incubated with secondary antibody conjugated to horseradish peroxidase, followed by signal detection using an enhanced chemiluminescence western blotting substrate (Bio-Rad, USA). The primary antibodies used here are listed in Supplementary Table 2.

### Xenograft assay

Four-week-old male nude mice were subcutaneously injected with 1 × 10^7^ UPK1A-AS1-overexpressing or negative control MHCC-97H cells. Tumor diameters were measured every other day and tumor volumes were calculated as (length × width^2^)/2. The mice were sacrificed before tumor removal 4 weeks after injection. All animal study procedures were approved by the Animal Use and Care Committee of Nanfang Hospital, Southern Medical University (Guangzhou, China).

### Functional enrichment analysis

A total of 500 genes positively correlated with EZH2 in HCC samples from TCGA datasets were subjected to functional enrichment analysis using the online software Metascape (https://metascape.org/). Only terms with a *P* < 0.01, minimum count of 3, and enrichment factor greater than 1.5 were identified as significant.

### RNA Immunoprecipitation (RIP)

The RIP assay was performed using the Magna RIP RNA-Binding Protein Immunoprecipitation Kit (Millipore, USA) following the manufacturer’s recommendations. Cells were lysed with lysis buffer, and cell lysates were immunoprecipitated with anti-EZH2 and immunoglobulin (Ig) G antibodies. Immunoprecipitated RNA was extracted, purified, and reverse transcribed to cDNA. The transcribed cDNA was subjected to qRT-PCR using UPK1A-AS1-specific primers. The primer sequences used for UPK1A-AS1 amplification are listed in Supplementary Table 1.

### RNA pull-down assay

An RNA pull-down assay was carried out using a Magnetic RNA-Protein Pull-Down Kit (Pierce, USA) following the manufacturer’s instructions. Full-length UPK1A-AS1 and antisense were obtained using RiboMAX Large Scale RNA Production Systems (Promega, USA). Biotin-labeled UPK1A-AS1 and antisense were bound to the beads and incubated with whole-cell protein lysates for immunoprecipitation. The beads were washed before being eluted with SDS-PAGE loading buffer. Samples eluted from beads were subjected to western blotting analysis.

### Immunofluorescence (IF)

MHCC-97H cells overexpressing UPK1A-AS1 were seeded on coverslips for IF staining. Briefly, cells were washed with phosphate-buffered saline (PBS), fixed with 4% paraformaldehyde, permeabilized with 0.25% Triton-X-100, and blocked with 5% BSA at room temperature. Cells on the coverslips were incubated with anti-EZH2 (1:100) at 4 °C overnight, followed by incubation with Cy3-conjugated CA goat antibodies against rabbit IgG (Santa Cruz Biotechnology, USA) for 1 h at room temperature. The cells were further counterstained with 4, 6-diamidino-2-phenylindole (DAPI, Invitrogen, USA) before imaging with a microscope (Carl Zeiss LSM880, Germany).

### Fluorescence in Situ Hybridization Analysis (FISH)

The UPK1A-AS1 RNA FISH probe was designed and synthesized by Yeshan Bio (Guangzhou, China). In brief, cells on the coverslips were fixed with 4% paraformaldehyde for 20 min, washed with PBS twice, treated with pepsin, and pre-hybridized with hybridization reaction solution at 55 °C for 2 h. After pre-hybridization, 20 μL hybridization reaction solution containing 3 μM probes was added to the coverslips. Cells on the coverslips were hybridized at 37 °C overnight and then washed with 2 × saline sodium citrate at 37 °C, followed by washing with PBS twice at room temperature. Finally, cells on the coverslips were stained with DAPI before imaging with a microscope (Carl Zeiss LSM880).

### Luciferase reporter assay

The sequence of the full-length UPK1A-AS1 (UPK1A-AS1-wt) and miR-138-5p binding site mutation of UPK1A-AS1 (UPK1A-AS1-mut) were cloned and inserted into the psiCHEK-2.0 vector (Promega, USA). For the dual-luciferase reporter assay, MHCC-97H cells were co-transfected with miR-138-5p and UPK1A-AS1-wt or UPK1A-AS1-mut plasmids. Luciferase activity was measured 48 h after transfection using the Dual-Luciferase Reporter Assay System (Promega) according to the manufacturer’s instructions.

### Statistical methods

All statistical analyses were carried out using SPSS statistical software version 22 (Abbott Laboratories, USA). Student’s t-test and one-way ANOVAs were performed for statistical analysis when appropriate. Kaplan-Meier and log-rank tests were used for survival analysis. A *P*-value < 0.05 (two-tailed) was considered statistically significant.

## Results

### Upregulation of UPK1A-AS1 promotes proliferation of HCC cells

**Fig. 1:**
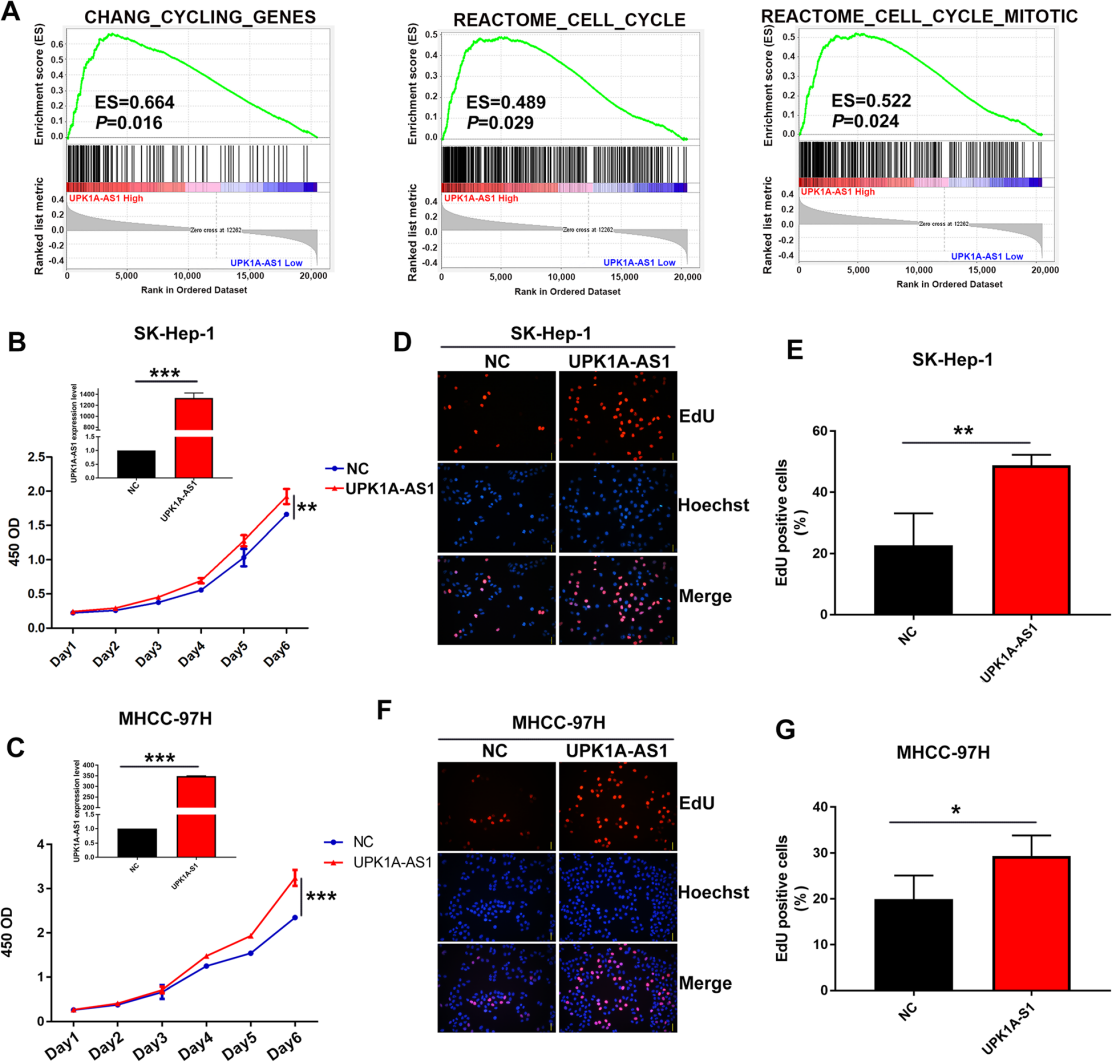
Upregulation of UPK1A-AS1 promotes proliferation in HCC cells. **a**. Results of Gene Set Enrichment Analysis (GSEA)were plotted to visualize the correlation between UPK1A-AS1 and cell cycle gene signatures in HCC sample from TCGA dataset (*P* < 0.05). **b-c**. CCK-8 assay was performed to determine the effect of UPK1A-AS1 overexpression on proliferation in SK-Hep-1 (**b**) and MHCC-97H (**c**) cells (^**^P < 0.01, ^***^P < 0.001). **d-g**. Overexpression of UPK1A-AS1 in SK-Hep-1 (**d-e**) and MHCC-97H (**f-g**) cells promoted more cells into S phase than negative control as detected by EdU assay (^*^P < 0.05, ^**^P < 0.01)

Detection of UPK1A-AS1 expression in HCC cell lines suggested that UPK1A-AS1 was highly expressed in HCC cells compared to the human hepatocyte cell line L02 (Supplementary Figure 1A). To investigate the molecular mechanism of UPK1A-AS1, we conducted GSEA of TCGA cohort and found that high UPK1A-AS1-expressing groups were enriched for cell cycle-related gene sets (Fig. 1a), suggesting that UPK1A-AS1 may have a role in cell proliferation. To confirm its effect on cell proliferation, lentiviral vectors with full-length UPK1A-AS1 or negative control were introduced into HCC cells, and the proliferation rate of HCC cells was examined. UPK1A-AS1 was successfully overexpressed in HCC cells, and the upregulation of UPK1A-AS1 significantly promoted HCC cell proliferation, as detected by CCK-8 assay (Fig. 1b–c). Since the upregulation of UPK1A-AS1 correlated with cell cycle-related gene sets, we further determined whether UPK1A-AS1 could affect HCC cell cycle progression. We then performed EdU dye assays to examine the change in the ratio of cells entering the S phase. The results showed that more UPK1A-AS1-overexpressing cells entered the S phase than the control cells (Fig. 1d–g). Collectively, UPK1A-AS1 overexpression promoted HCC proliferation.

### UPK1A-AS1 downregulation inhibits HCC cell proliferation

**Fig. 2:**
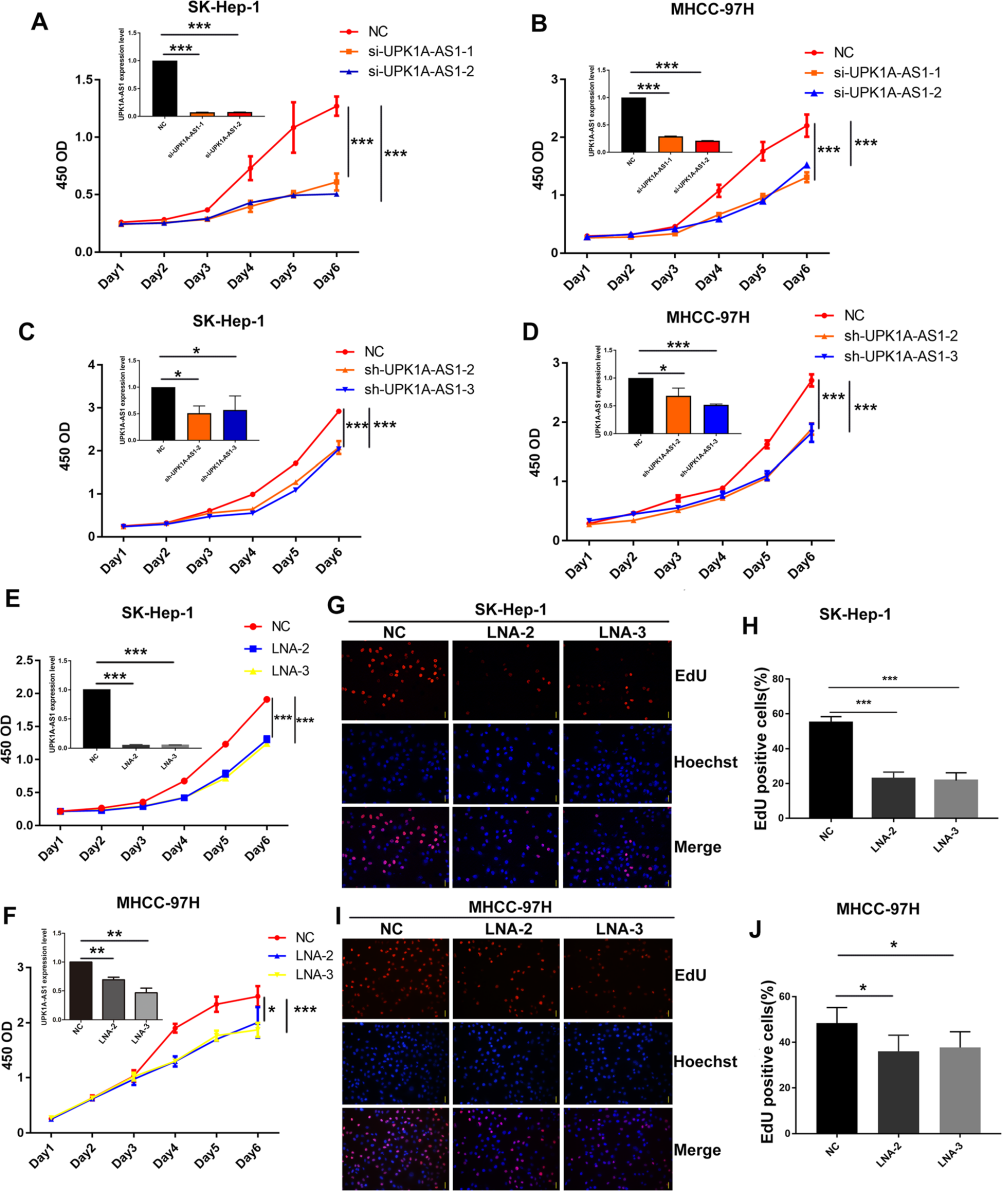
Knock down of UPK1A-AS1 inhibits proliferation in HCC cells. **a-b**. CCK-8 assay was performed to determine the effect of si-UPK1A-AS1 on proliferation in SK-Hep-1 (**b**) and MHCC-97H (**c**) cells (^***^P < 0.001). **c-d**. Proliferation curve of HCC cells transfected with sh-UPK1A-AS1 (^*^P < 0.05, ^***^P < 0.001). **e-f**. Representative CCK-8 analysis of cell viability after downregulation of UPK1A-AS1 by LNAs in SK-Hep-1(**e**) and MHCC-97H (**f**) cells (^*^P < 0.05, ^**^P < 0.01, ^***^P < 0.001). **g-j**. EdU assay showed that downregulation of UPK1A-AS1 decreased the ratio of S phase cells in SK-hep-1(**g-h**) and MHCC-97H (**i-j**) cells (^*^P < 0.05)

To further confirm the regulatory function of UPK1A-AS1 on cell proliferation, we knocked down UPK1A-AS1 expression in HCC cells using siRNAs and shRNAs. The knockdown efficiency was verified using qRT-PCR (Fig. 2a–d). CCK-8 assays showed that the downregulation of UPK1A-AS1 visibly inhibited HCC cell proliferation (Fig. 2a–d). Specific locked nucleic acids (LNAs) targeting UPK1A-AS1 were introduced into HCC cells to further verify the effect of UPK1A-AS1 downregulation on HCC proliferation. Consistently, the downregulation of UPK1A-AS1 by LNAs also impaired HCC proliferation (Fig. 2e-f). Moreover, cells that entered the S phase in the LNA treatment groups were significantly less than those in the control groups (Fig. 2g–j).

To address the possibility of off-target effects, an overload of UPK1A-AS1 was introduced into HCC cells pre-subjected to UPK1A-AS1 downregulation. Overload of UPK1A-AS1 successfully rescued the expression of UPK1A-AS1 downregulated by siRNAs specifically targeting UPK1A-AS1 (Supplementary Figure 1B–C). Notably, the downregulation of UPK1A-AS1 by specific siRNAs significantly inhibited HCC cell proliferation, and this effect was eliminated by the restored expression of UPK1A-AS1, as detected by CCK-8 and EdU assays (Supplementary Figure 1B–G). In summary, knockdown of UPK1A-AS1 inhibited HCC cell proliferation.

### UPK1A-AS1 accelerates the G1/S transition of HCC cells

**Fig. 3:**
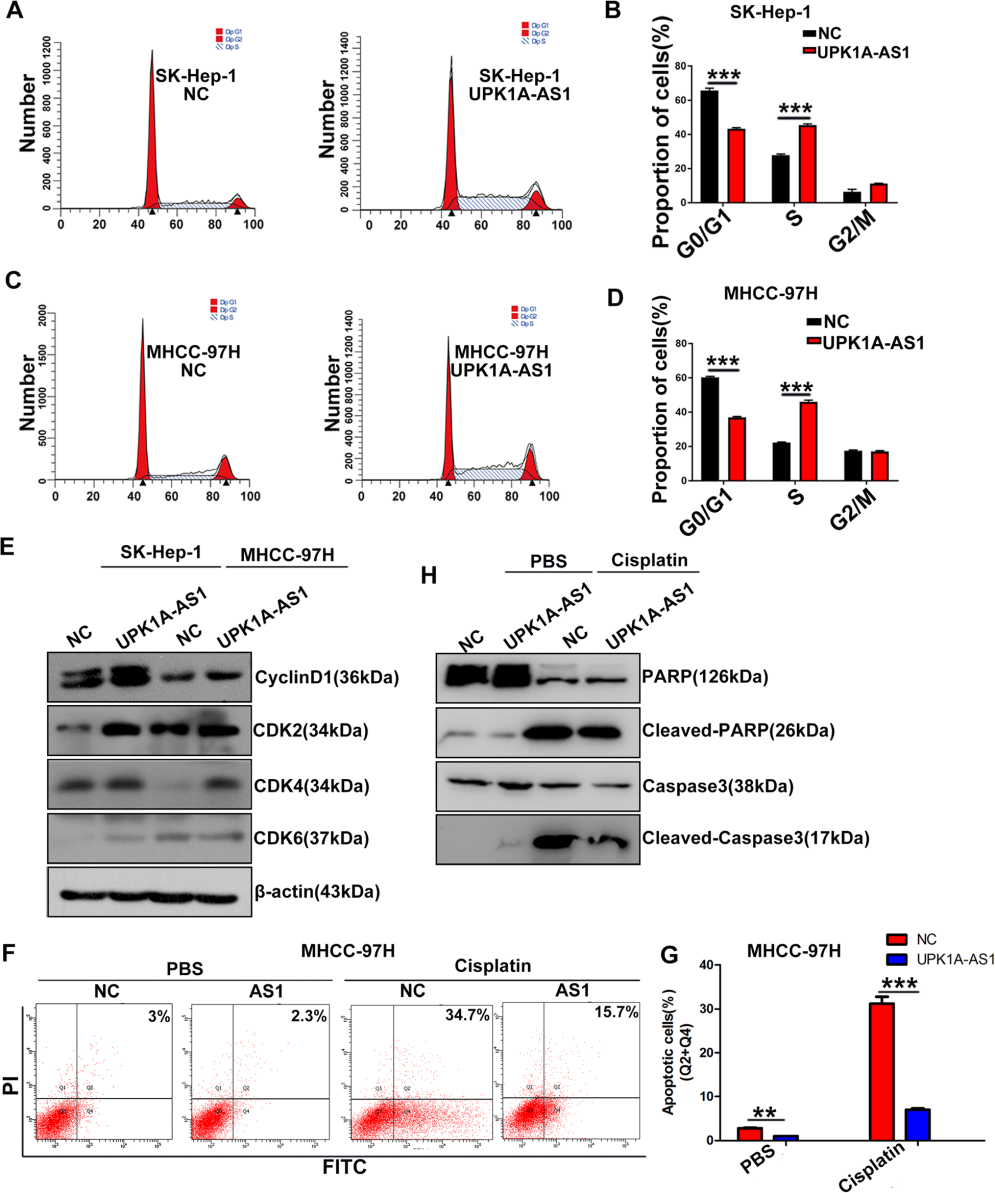
UPK1A-AS1 promotes the G1/S transition of HCC cells. **a-d.** UPK1A-AS1 increased the ration of cells in S phase in comparison with the negative control in SK-Hep-1 (**a-b**) and MHCC-97H (**c-d**) cells (^***^P < 0.001). Error bars represent the mean ± S.D. of three independent experiments. **e**. UPK1A-AS1 increased expression of CyclinD1, CDK2, CDK4 and CDK6 in HCC cells by western blotting. **f**. UPK1A-AS1-overexpressing cells treated with cis-platinum (40 μM) for 24 h. The cells stained with Annexin V-FITC and PI were subjected to FACS analysis. Error bars represent the mean ± S.D. of triplicate experiments (^**^P < 0.01, ^***^P < 0.001). **h**. The expression of caspase-3 and PARP were analyzed using western blottinh in UPK1A-AS1-overexpressing MHCC-97H cells treated with cis-platinum

It is well accepted that rapid cell cycle progression accounts for cancer proliferation. The above results showed that the upregulation of UPK1A-AS1 correlated with cell cycle-related gene sets, and promoted cell proliferation. This led us to hypothesize that UPK1A-AS1 might regulate cell cycle progression. To this end, we carried out flow cytometry analyses to detect the distribution of cell cycle phases in HCC cells following UPK1A-AS1 overexpression or downregulation. The results showed that HCC cells with UPK1A-AS1 overexpression had a decreased rate of G1 phase cells and an increased rate of S phase cells (Fig. 3a–d). Consistently, CyclinD1, CDK2, CDK4, and CDK6, important modulators of G1/S transition, were highly expressed in cells overexpressing UPK1A-AS1 (Fig. 3e, Supplementary Figure 2B). However, the expression of CyclinE1, p21, and p27 remained unchanged in cells overexpressing UPK1A-AS1 (Supplementary Figure 2A-B). In contrast, si-UPK1A-AS1 resulted in an evident cell cycle arrest at the G1/G0 phase (Supplementary Figure 2C–D), and CyclinD1, CDK2, CDK4, and CDK6 were visibly decreased in cells with UPK1A-AS1 downregulation (Supplementary Figure 2E–F).

We also explored the effect of UPK1A-AS1 on apoptosis and drug resistance. Lower levels of apoptosis were found in UPK1A-AS1-overexpressing cells, indicating that overexpression of UPK1A-AS1 could protect HCC cells from cis-platinum toxicity (Fig. 3f–g). Consistently, the expression levels of several well-defined apoptosis markers, including cleaved caspase3 and cleaved PARP, were markedly decreased in UPK1A-AS1-overexpressing cells after cis-platinum exposure (Fig. 3h, Supplementary Figure 2G), suggesting that UPK1A-AS1 may boost the resistance to chemotherapy with cis-platinum in HCC cells. In conclusion, the upregulation of UPK1A-AS1 accelerated the G1/S transition of HCC cells.

### UPK1A-AS1 promotes tumor growth in vivo

**Fig. 4:**
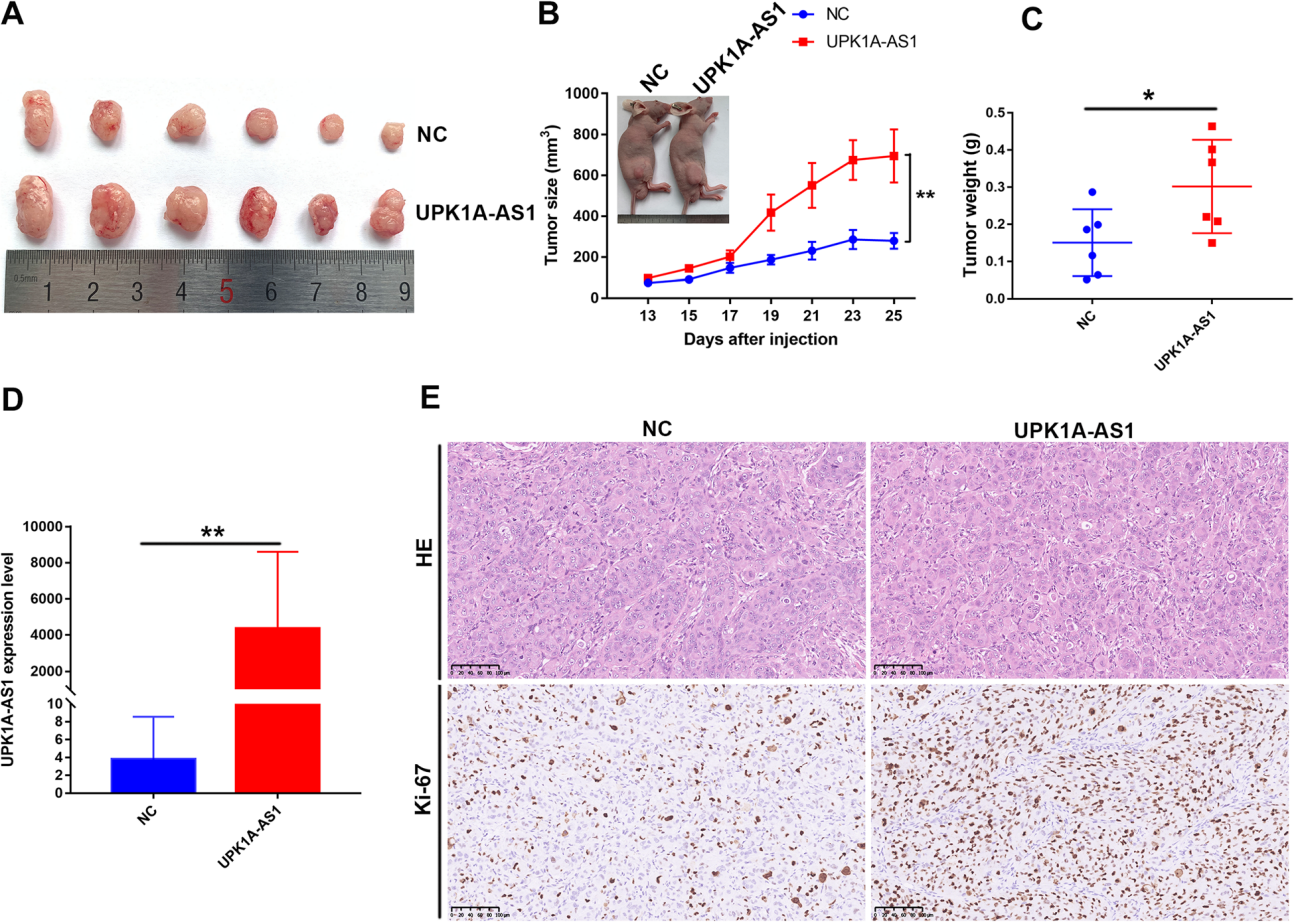
Overexpression of UPK1A-AS1 boosts tumor growth in HCC. **a-c**. Overexpression of UPK1A-AS1 in MHCC-97H cells promoted tumor growth in vivo. Tumor burden and tumor weight in cells with UPK1A-AS1 overexpression were visibly higher than those of control cells (^*^P < 0.05, ^**^P < 0.01). **d**. Expression of UPK1A-AS1 in the subcutaneous tumor was determined by qRT-PCR (^**^P < 0.01). **e**. Sections of xenograft tumors stained with hematoxylin and eosin (**h&e**); Ki-67 staining was performed for further determination of the effect of UPK1A-AS1 on cell proliferation

Based on our in vitro UPK1A-AS1 findings, we speculated that it might play an important role in tumor growth in vivo. HCC cells with stable UPK1A-AS1-overexpressing or negative controls were then subcutaneously injected into nude mice. The tumors formed in the UPK1A-AS1-overexpressing group (*n* = 6) grew faster than those in the negative control group (*n* = 6). The tumor weight and volume were significantly higher in the UPK1A-AS1-overexpressing group than in the negative control group (Fig. 4a–c, Supplementary Figure 3A). UPK1A-AS1 was remarkably overexpressed in the UPK1A-AS1-overexpressing group, as detected by qRT-PCR (Fig. 4d). In addition, the positive rate of the proliferation marker Ki-67 was obviously increased in tumors with UPK1A-AS1-overexpressing cells (Fig. 4e). Collectively, UPK1A-AS1 boosted tumor growth in vivo.

### UPK1A-AS1 correlates with EZH2-mediated cell cycle progression

**Fig. 5:**
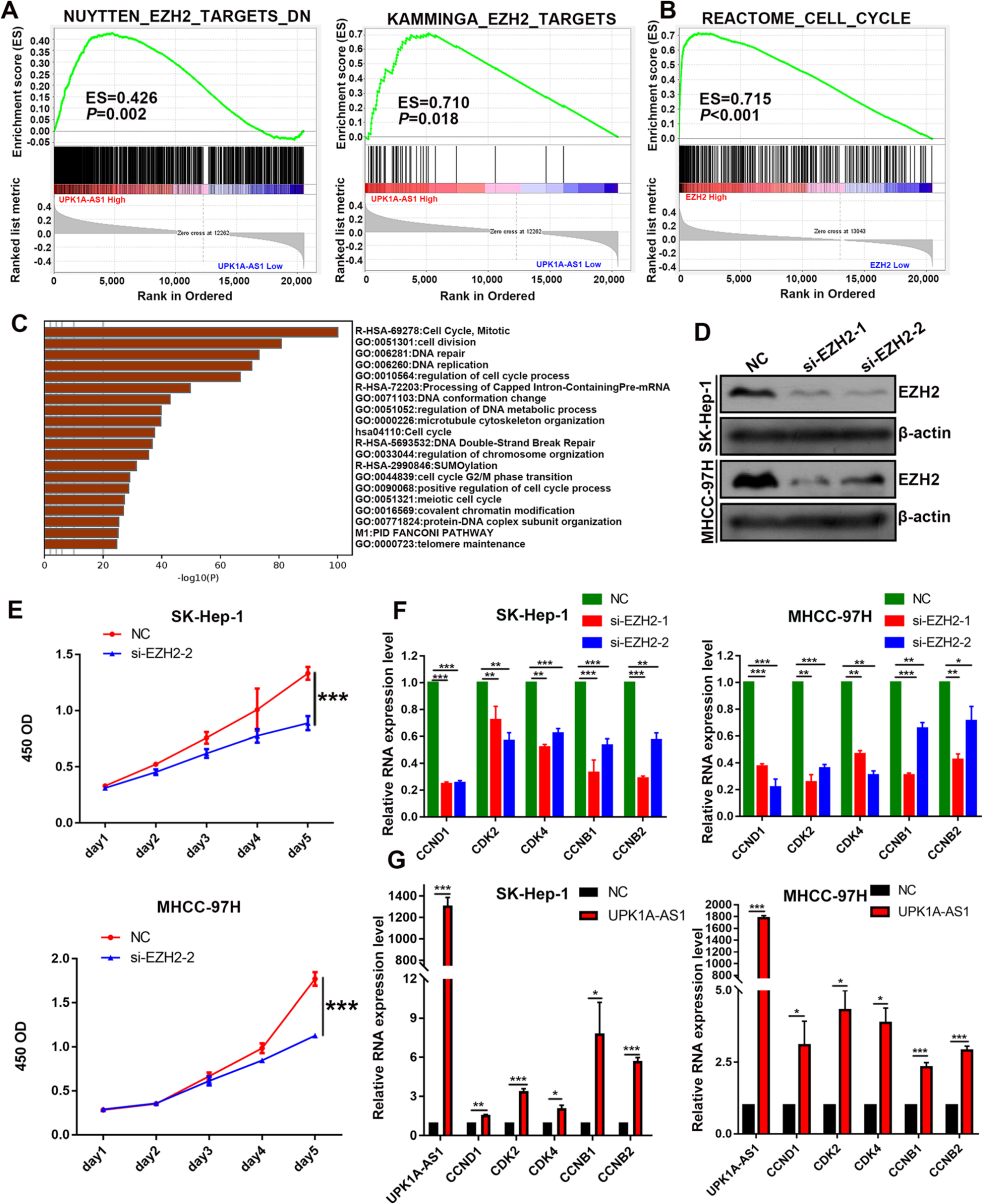
UPK1A-AS1 correlates with EZH2 targets. **a**. GSEA analysis plot indicated a significant correlation between UPK1A-AS1 and EZH2 targets (*P* < 0.05). **b**. Visualization of GSEA findings of the correlation between EZH2 and cell cycle gene signatures in HCC sample from TCGA dataset (*P* < 0.05). **c**. Heatmap of Gene Ontology (GO) enriched terms colored by *P*-values. **d**. Knockdown efficiency of si-EZH2 in SK-Hep-1 and MHCC-97H cells was measured by western blotting. **e**. Growth curve of HCC cells transfected with si-EZH2 (^***^P < 0.001). **f**. RNA expression of the indicated genes after si-EZH2 was measured using qRT-PCR (^*^P < 0.05, ^**^P < 0.01, ^***^P < 0.001). **g**. Overexpression of UPK1A-AS1 increased the expression of the indicated genes measured by qRT-PCR (^*^P < 0.05, ^**^P < 0.01, ^***^P < 0.001)

To dissect the molecular mechanism involved in UPK1A-AS1-associated HCC progression, GSEA was carried out with HCC tumor samples in TCGA datasets. GSEA results suggested that high expression of UPK1A-AS1 was correlated with EZH2 targets (Fig. 5a). Interestingly, the GSEA results showed that high EZH2 expression was positively correlated with cell cycle gene sets (Fig. 5b). Additionally, the functions of EZH2 and its correlated genes in HCC were predicted by analyzing Gene Ontology (GO) and Kyoto Encyclopedia of Genes and Genomes pathways in Metascape. The top 20 GO enrichment items also suggested that EZH2 was associated with the cell cycle (Fig. 5c). It has been long recognized that EZH2 plays a crucial role in regulating cancer cell proliferation [17]. Consistent with previous studies, EZH2 downregulation with siRNA significantly inhibited HCC cell proliferation (Fig. 5d–e, Supplementary Figure 2B). CCND1, CDK2, and CDK4, which accelerate cell cycle progression, were reported to be downstream targets of EZH2 [18]. Not surprisingly, these genes were significantly downregulated after EZH2 silencing in HCC cells (Fig. 5f, Supplementary Figure 3C–D). We also found that CCNB1 and CCNB2 were significantly decreased after EZH2 silencing in HCC cells (Fig. 5f, Supplementary Figure 3C-D). These results suggested that CCND1, CDK2, CDK4, CCNB1, and CCNB2 were direct targets of EZH2 in HCC. We further investigated the correlation between EZH2 and its targets from TCGA datasets. Strong positive correlations between EZH2 and CCND1, CDK2, CDK4, CCNB1, and CCNB2 were found in HCC samples (Supplementary Figure 4A), suggesting that EZH2 promoted HCC proliferation by regulating cell cycle-related genes.

Given that UPK1A-AS1 was correlated with the EZH2 target, and both regulated HCC proliferation, we speculated that UPK1A-AS1 boosted HCC cell progression by regulating these cell cycle-related EZH2 targets. As expected, the upregulation of UPK1A-AS1 significantly increased the expression of EZH2 targets, including CCND1, CDK2, CDK4, CCNB1, and CCNB2 (Fig. 5g, Supplementary Figure 4C–D). Furthermore, positive correlations between UPK1A-AS1 and CDK2, CDK4, CCNB1, and CCNB2, except for CCND1, were found in HCC samples (Supplementary Figure 4B), indicating that UPK1A-AS1 regulated CCND1 in a more complicated manner. In short, these results indicated that UPK1A-AS1 regulated EZH2-mediated cell cycle progression.

### UPK1A-AS1 interacts with EZH2

**Fig. 6:**
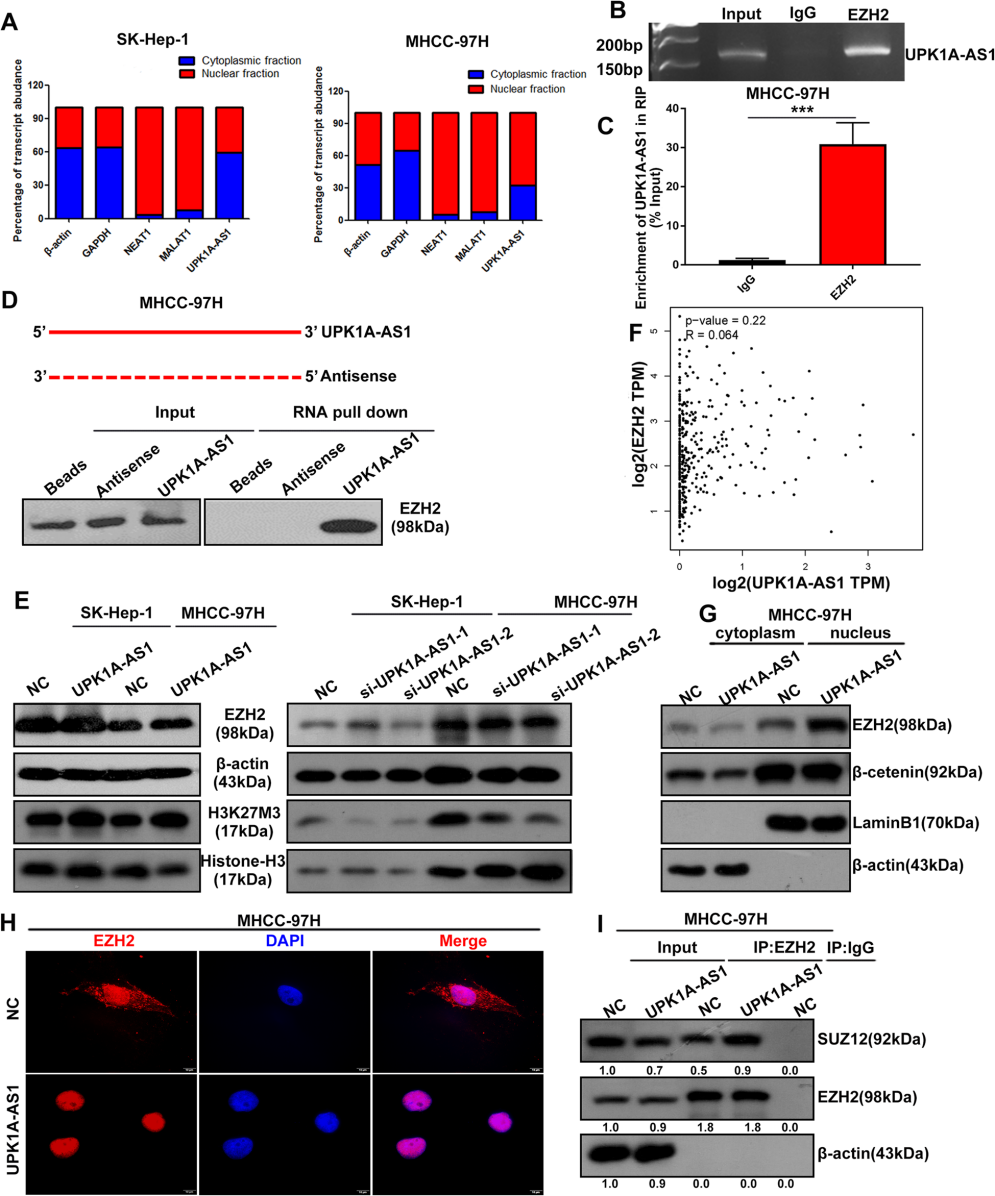
UPK1A-AS1 interacts with EZH2. **a**. UPK1A-AS1 cytosolic and nuclear expression levels in SK-Hep-1 and MHCC-97H cells. β-Actin and U6 were used as cytosolic and nuclear markers, respectively. Both NEAT1 and MALAT1 were localized to the nucleus. **b-c**. RIP assay was performed in MHCC-97H cells and the co-precipitated RNA was subjected to qRT-PCR for UPK1A-AS1(^***^P < 0.001). **d**. RNA pull-down assay was carried out to confirm the association between UPK1A-AS1 and EZH2. **e**. Effect of UPK1A-AS1 overexpression on EZH2 and H3K27M3 expressions was measured by western blotting. **f**. Correlation between UPK1A-AS1 and EZH2 was analyzed in HCC samples from TCGA dataset. **g**. EZH2 expression level in the cytoplasm or nucleus of MHCC-97H cells. β-Actin was used as a cytosol marker, LaminB1 served as a nuclear marker. **h**. Translocation of EZH2 from the cytoplasm to the nucleus was detected by immunofluorescence assay. **i**. Immunoprecipitation assay identified the increased interaction between EZH2 and SUZ12 in UPK1A-AS1-overexpressing MHCC-97H cells. β-Actin was used as negative control

To further investigate the molecular mechanisms by which UPK1A-AS1 contributes to the progression of HCC, we examined the subcellular distribution of UPK1A-AS1 in HCC cells by fractionation and FISH assays. UPK1A-AS1 was localized to both the nucleus and cytosol of HCC cells, indicating that it could function as a modulator of gene transcription (Fig. 6a, Supplementary Figure 5A). It has been reported that one-fifth of human lncRNAs physically interact with polycomb repressive complex 2 (PRC2), consisting of EZH2, SUZ12, and EED, among which EZH2 is highlighted as a crucial component of PRC2 [19]. We showed that UPK1A-AS1 regulated EZH2-mediated cell cycle progression, suggesting that UPK1A-AS1 may interact with and bind to EZH2. To test our hypothesis, an RIP assay against EZH2 was performed. The RIP assay showed that UPK1A-AS1 was significantly enriched with the EZH2 antibody compared with that of the negative control (IgG) in HCC cells (Fig. 6b–c). To further confirm our assumption, the interaction of UPK1A-AS1 with EZH2 was determined using an RNA pull-down assay. The results showed that biotin-labeled UPK1A-AS1, but not antisense, exhibited the ability to harbor EZH2 protein (Fig. 6d). These results demonstrated that UPK1A-AS1 could physically interact with EZH2.

We then wondered whether UPK1A-AS1 had an impact on the expression level of EZH2. Western blotting assay showed that neither overexpression nor downregulation of UPK1A-AS1 altered the expression of EZH2 (Fig. 6e, Supplementary Figure 5B). Moreover, no significant correlation was found between UPK1A-AS1 and EZH2 expression levels (Fig. 6f). These results demonstrated that UPK1A-AS1 interacted with EZH2 without changing the expression of EZH2. Surprisingly, the overexpression of UPK1A-AS1 increased the trimethylation of H27K3, which was caused by PRC2 activation. In contrast, silencing UPK1A-AS1 led to an obvious reduction of trimethylation on H27K3 (Fig. 6e, Supplementary Figure 5B), suggesting that the interaction between UPK1A-AS1and EZH2 led to PRC2 activation.

It has been reported that lncRNAs physically interact with and bind to proteins to alter their subcellular distribution [20]. Fractionation assays showed that the overexpression of UPK1A-AS1 decreased the cytoplasmic expression of EZH2 but increased the expression level of EZH2 in the nucleus (Fig. 6g, Supplementary Figure 5C). IF experiments also confirmed that overexpression of UPK1A-AS1 induced the translocation of EZH2 from the cytoplasm to the nucleus (Fig. 6h). EZH2, SUZ12, and EED form a complex in the nucleus for PRC2 activation. An increased interaction between EZH2 and SUZ12 was found after UPK1A-AS1 overexpression (Fig. 6i). In brief, UPK1A-AS1 interacted with EZH2, mediated its nuclear translocation, and reinforced its binding to SUZ12, leading to increased trimethylation of H27K3.

### UPK1A-AS1 functions through EZH2

**Fig. 7:**
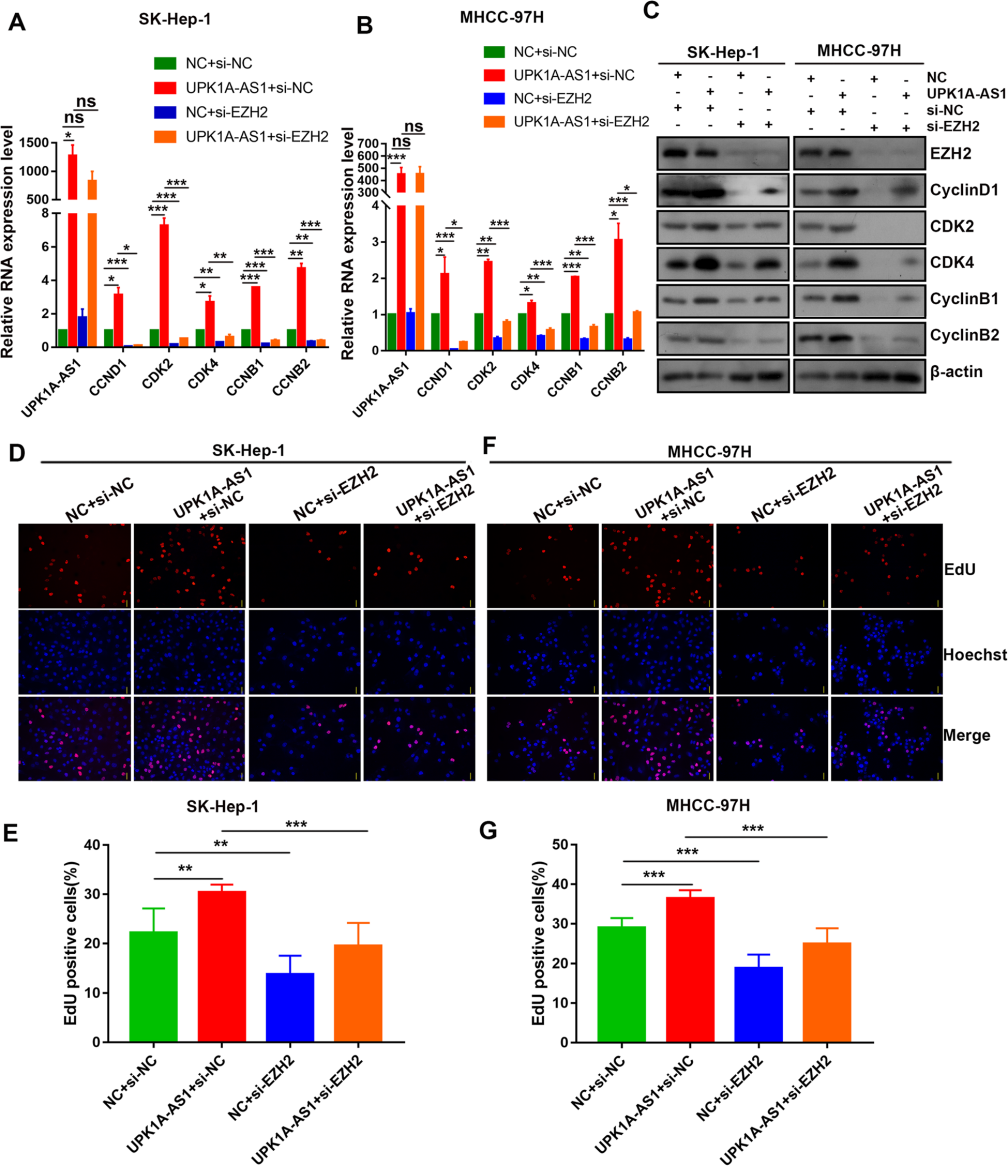
UPK1A-AS1 functions through EZH2. **a-b**. Expression of the indicated genes were monitored by qRT-PCR after co-transfection of UPK1A-AS1 vectors and si-EZH2 into SK-Hep-1 (**a**) and MHCC-97H (**b**) cells (^*^P < 0.05, ^**^P < 0.01, ^***^P < 0.001). **c**. Expression of the indicated proteins was measured by western blotting after co-transfection of UPK1A-AS1 vectors and si-EZH2 into SK-Hep-1 and MHCC-97H cells. **d-g**. Effect of co-transfection of UPK1A-AS1 vectors and si-EZH2 on the cell proliferation of SK-Hep-1 (**d-e**) and MHCC-97H (**f-g**) cells, as measured by the EdU assay (^**^P < 0.01, ^***^P < 0.001)

To explore whether EZH2 mediated the regulative effect of UPK1A-AS1 on HCC cell proliferation, we co-transfected EZH2 siRNA and UPK1A-AS1 vectors into HCC cells and analyzed the expression of EZH2 targets related to the cell cycle. Overexpression of UPK1A-AS1 increased the expression of CCND1, CDK2, CDK4, CCNB1, and CCNB2, which was eliminated by downregulation of EZH2 (Fig. 7a–c, Supplementary Figure 5D). Consistent with the results of qRT-PCR, the EdU assay showed that more UPK1A-AS1-overexpressing cells entered the S phase than the control cells. The increase in the S phase ratio by UPK1A-AS1 overexpression was partly reversed by silencing EZH2 (Fig. 7d–g). Taken together, targeting EZH2 with specific siRNA impaired the UPK1A-AS1-mediated upregulation of proliferation and cell cycle progression related genes.

### UPK1A-AS1 promotes HCC cell proliferation partially by sponging miR-138-5p

It is well recognized that cytoplasmic lncRNAs can bind to miRNAs and function as sponges to modulate the availability of miRNAs for binding with their target mRNAs. Given that UPK1A-AS1 was located in both the nucleus and cytosol of HCC cells (Fig. 6a, Supplementary Figure 5A), we speculated that cytoplasmic UPK1A-AS1 might directly interact with miRNAs. A putative miR-138-5p binding site was identified within the UPK1A-AS1 sequence, as predicted by miRcode (http://mircode.org/index.php) online software (Supplementary Figure 6A). To test whether miR-138-5p recognized the predicted binding site within UPK1A-AS1, we constructed luciferase activity reporter vectors of wild type and mutant UPK1A-AS1 (the binding motif for miR-138-5p was mutated, Supplementary Figure 6A). Dual-luciferase assays showed that overexpression of miR-138-5p significantly decreased the relative luciferase activity of reporter vectors containing UPK1A-AS1-wt but that with UPK1A-AS1-mut remained unchanged (Supplementary Figure 6B).

Accumulating evidence indicates that miRNAs bind to their target mRNAs and cause RNA degradation, translation suppression, or both by forming an RNA-induced silencing complex (RISC) with Argonaute 2 (AGO2). To test whether endogenous UPK1A-AS1 existed in miRNA-containing RISC, an RIP assay against the AGO2 antibody was employed in HCC cells. Both UPK1A-AS1 and miR-138-5p were enriched in the AGO2-associated complex but not in the control IgG (Supplementary Figure 6C–D), indicating that miR-138-5p is a bona fide UPK1A-AS1-targeting miRNA. Collectively, these results suggested that UPK1A-AS1 physically bound to miR-138-5p and might act as a sponge for miR-138-5p.

miR-138-5p targets multiple cell cycle-related genes, including CDK6, which play an important role in the G1/S transition. We wondered whether miR-138-5p regulated CDK6 expression in HCC cells. Overexpression of miR-138-5p markedly decreased both mRNA and protein expression of CDK6, as detected by qRT-PCR and western blotting assays (Supplementary Figure 6 E–G). Given that the overexpression of UPK1A-AS1 considerably increased the expression of CDK6, we hypothesized that UPK1A-AS1 regulated CDK6 expression by sponging miR-138-5p. As expected, the upregulation of UPK1A-AS1 induced CDK6 expression. However, overexpression of miR-138-5p downregulated the expression of CDK6, while UPK1A-AS1 induction partly reversed this result (Supplementary Figure 6 H–I).

To determine whether the proliferation promotion effect of UPK1A-AS1 was mediated by miR-138-5p, HCC cells with UPK1A-AS1-overexpressing or negative control were co-transfected with miR-138-5p mimic or miR-NC. EdU experiments demonstrated that miR-138-5p overexpression increased cell proliferation induced by UPK1A-AS1 overexpression (Supplementary Figure 6 J–M). In summary, these results suggested that UPK1A-AS1 promoted HCC cell proliferation partially by sponging miR-138-5p.

### High expression of UPK1A-AS1 predicts poor prognosis for patients with HCC

UPK1A-AS1 is a newly identified lncRNA, and little is known about its clinical implication in cancers. Genotype-Tissue Expression (GTEx) benign tissue RNA-seq revealed that UPK1A-AS1 was highly expressed in the bladder but scarcely in other tissues (Supplementary Figure 7A). However, data from TCGA datasets showed that UPK1A-AS1 was relatively induced in some types of cancers, including HCC (Supplementary Figure 7B), indicating its important role in the development and progression of malignancies.

**Fig. 8:**
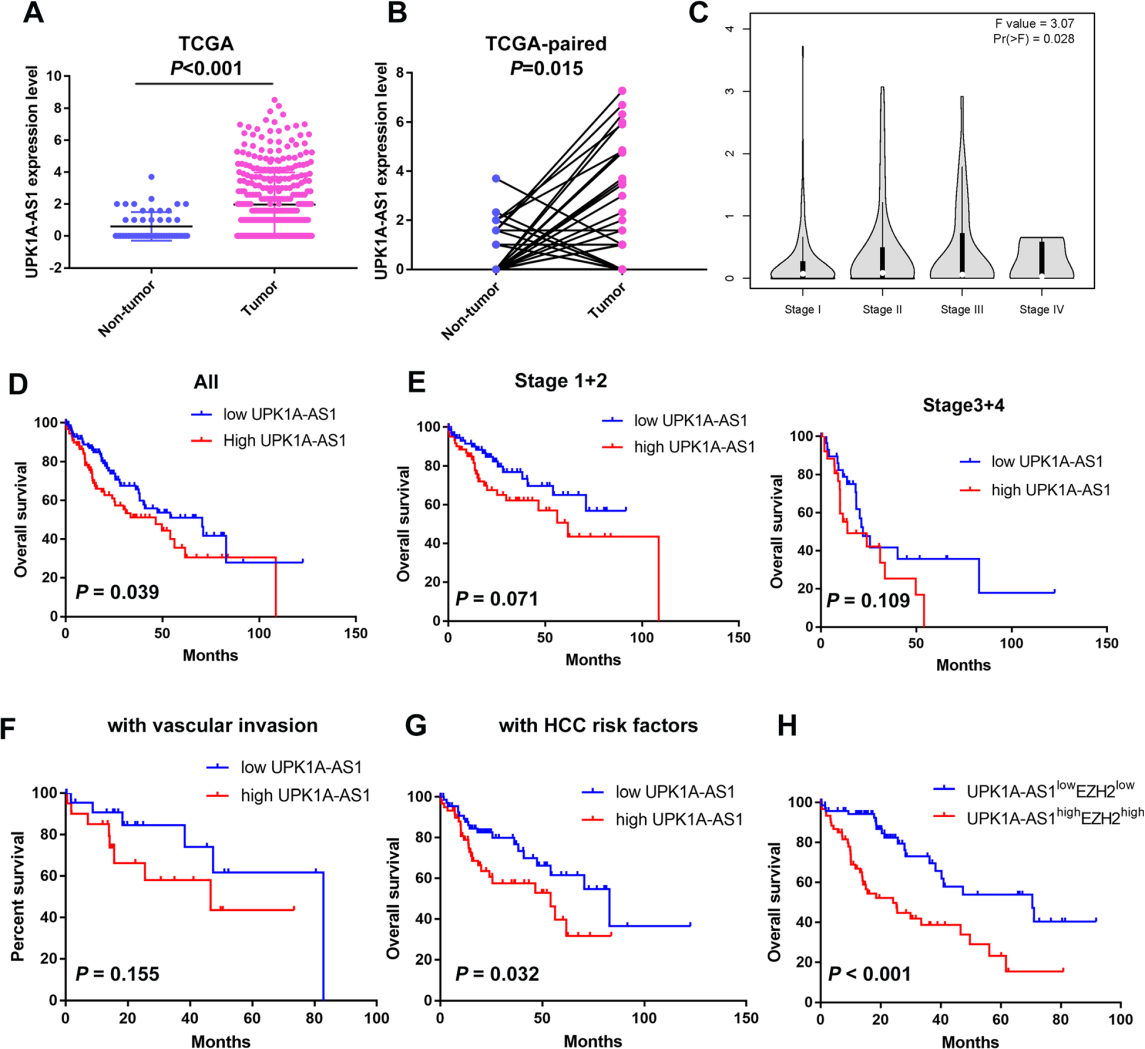
Upregulation of UPK1A-AS1 correlates with poor prognosis in patients with HCC. **a**. Expression level of UPK1A-AS1 in HCC from TCGA dataset (*P* < 0.001). **b**. The expression of UPK1A-AS1 from 50 paired HCC samples and adjacent non-tumor liver tissues from TCGA dataset (*P* < 0.05). **c**. Correlation between UPK1A-AS1 and tumor stage in patients with HCC (GEPIA). **d**. Kaplan-Meier analysis of overall survival in the TCGA dataset based on UPK1A-AS1 expression (*P* < 0.05). **e-g**. Kaplan-Meier analysis showed the correlation of UPK1A-AS1 and survival in indicated groups in HCC from TCGA dataset. **h**. Kaplan-Meier analysis of overall survival in the TCGA dataset based on UPK1A-AS1 and EZH2 expression levels (*P* < 0.001)

To elucidate the clinical implication of UPK1A-AS1 in HCC, UPK1A-AS1 expression levels in HCC were analyzed using RNA-seq data from TCGA datasets. UPK1A-AS1 was highly expressed in HCC cells (Fig. 8a). To eliminate the possible contribution of the imbalanced sample size to the statistical significance, we continued to compare the expression of UPK1A-AS1 in paired HCC and corresponding non-tumor samples. The results confirmed that UPK1A-AS1 was significantly overexpressed in HCC (Fig. 8b). Moreover, the high expression of UPK1A-AS1 was positively correlated with the tumor stage of HCC (Fig. 8c). Survival analysis showed that patients with high expression of UPK1A-AS1 exhibited worse overall survival (OS) than those with low UPK1A-AS1 expression (Fig. 8d). As UPK1A-AS1 expression correlated with the HCC stage, we reanalyzed the data from subgroups. Patients with high levels of UPK1A-AS1 presented shorter OS than those with low UPK1A-AS1 expression, although the difference did not reach statistical significance (Fig. 8e). Vascular invasion is a sign of poor prognosis in patients with HCC. Survival analysis showed that in the vascular invasion group, patients with high levels of UPK1A-AS1 suffered poorer OS. Due to limitations in sample size, the difference did not reach statistical significance (Fig. 8f). Since infection with hepatitis virus and alcohol abuse were risk factors for HCC, we also clarified the correlation between UPK1A-AS1 expression level and prognosis in patients with HCC risk factor exposure. It is shown that patients with high UPK1A-AS1 expression suffered shortened OS in patients with HCC risk factors (Fig. 8g). Furthermore, univariate Cox regression analysis identified UPK1A-AS1 as a risk factor for OS in patients with HCC (Table 1).

**Table 1:** Univariate and multivariate analyses of OS in TCGA cohort by Cox regression analysis

Variables	Univariate analysis			Multivariate analysis		
	HR	CI (95%)	*P* value	HR	CI (95%)	*P* value
Age(years)	1.502	0.821–2.748	0.285			
Gender	1.364	0.742–2.506	0.185			
Race	1.165	0.690–1.966	0.556			
Histologic grade	1.687	1.023–2.783	0.124			
HCC risk factor	0.472	0.263–0.848	0.015*	0.639	0.410–0.995	0.048*
Stage	2.552	1.584–4.112	0.000*	2.214	1.408–3.481	0.001*
Vascular invasion	0.561	0.336–0.936	0.120			
AFP	0.560	0.304–1.035	0.704			
New tumor event after initial treatment	1.035	0.628–1.707	0.631			
UPK1A-AS1	2.043	1.299–3.397	0.036*	1.704	1.100–2.640	0.017*

*Abbreviations*: *HBV* hepatitis B virus, *HCV* hepatitis C virus, *CI* confidence interval, *HR* hazard radio

*The values had statistically significant differences

We also explored the clinical significance of EZH2 in cancer. Data from TCGA datasets showed that EZH2 was highly expressed in various cancers, including HCC (Supplementary Figure 8A). EZH2 overexpression predicted poor prognosis in various cancers, suggesting its oncogenic role in tumorigenesis (Supplementary Figure 8B). A series of HCC datasets from the Gene Expression Omnibus confirmed that EZH2 was highly expressed in HCC (Supplementary Figure 8C). Moreover, high EZH2 expression correlated with the development and progression of HCC (Supplementary Figure 8 D–G). Survival analysis showed that EZH2 predicted poor prognosis for patients with HCC (Supplementary Figure 9A, C). Nonetheless, in patients undergoing sorafenib treatment, EZH2 was a factor affecting their survival (Supplementary Figure 9B). Furthermore, high expression of EZH2 was associated with poor prognosis in patients with vascular invasion (Supplementary Figure 9D). EZH2 was also potent in clarifying prognosis in patients with hepatitis virus and alcohol consumption (Supplementary Figure 9 E–F). Our results showed that UPK1A-AS1 functioned through EZH2, at least in part. Consistently, patients with simultaneous high UPK1A-AS1 and EZH2 expression also exhibited shorter OS (Fig. 8h). Collectively, UPK1A-AS1 was significantly upregulated in HCC, and the upregulation of UPK1A-AS1 predicted poor prognosis in patients with HCC.

## Discussion

Despite the profound advances made in HCC therapeutic strategies, the long-term prognosis of HCC patients remains poor due to limited understanding of the underlying mechanisms of tumor initiation and development [21]. Dysregulation of lncRNAs is involved in the onset and progression of malignancies, suggesting their clinical potential as biomarkers for diagnosis and prognosis, as well as therapeutic targets. Here, we demonstrated that UPK1A-AS1 was highly expressed in HCC, and high expression of UPK1A-AS1 predicted poor prognosis in patients with HCC. Biological experiments showed that UPK1A-AS1 promoted proliferation and tumor growth by accelerating the G1/S transition of HCC cells. Furthermore, we also found that overexpression of UPK1A-AS1 could protect HCC cells against cis-platinum toxicity, suggesting that UPK1A-AS1 may promote resistance to chemotherapy in HCC cells. Our findings suggest that UPK1A-AS1 may serve as a novel prognostic biomarker and a potential therapeutic target for HCC.

Little is known about the functional role and clinical significance of UPK1A-AS1 in cancers. UPK1A-AS1, downregulated in ESCC, inhibites the proliferation, migration, and invasion of ESCC cells by serving as a miRNA decoy [16]. In contrast, our findings showed that UPK1A-AS1 was upregulated in HCC, and the overexpression of UPK1A-AS1 promoted proliferation by regulating cell cycle progression. It is well accepted that lncRNAs consistently act in a tissue- or disease-specific manner [22]. RNA-seq data from GTEx revealed that UPK1A-AS1 was highly expressed in the bladder and modestly expressed in the esophagus, cervix, and vagina but hardly expressed in other tissues (Supplementary Figure 7A), indicating that the expression of UPK1A-AS1 was tissue-specific. Thus, the biological role of UPK1A-AS1 may vary depending on the organic context. The tissue- or disease-specific context of UPK1A-AS1 may account for the distinct roles of UPK1A-AS1 in ESCC and HCC.

Given the subcellular localization of lncRNAs in both the cytoplasm and nucleus, they utilize multiple molecular mechanisms to modulate protein function and gene activity [23]. Cytoplasmic lncRNAs can act as competing endogenous RNAs to interact directly with and sponge miRNA [24]. For example, lncRNA-HGBC regulates gallbladder cancer progression by interacting with miR-502-3p, thus sequestering miR-502-3p and downregulating SET expression [25]. SNHG1 specifically interacts with miR-154-5p, resulting in the induced expression of CCND2 [26]. In concert with these findings, our study demonstrated that lncRNA UPK1A-AS1 interactd with miR-138-5p, which is reported to be a tumor suppressor in HCC. Notably, miR-138-5p was downregulated in HCC and acted as a tumor suppressor to inhibit several cell cycle-related genes such as *CDK6* [27–29]. Indeed, UPK1A-AS1-induced CDK6 overexpression was partially dependent on the activity of miR-138-5p, as overexpression of miR-138-5p ablated UPK1A-AS1 activity in HCC. Given that UPK1A-AS1 also interacts with and sequesters other miRNAs (e.g., miR-1248) not limited to miR-138-5p, it is worthwhile to explore other miRNAs that potentially accelerate the growth of HCC during progression.

EZH2 serves as the core enzymatic subunit of PRC2, a complex that can methylate lysine 27 of histone H3 and facilitates chromatin remodeling and transcriptional silencing [30]. A growing body of evidence has implicated the oncogenic role of EZH2 in the progression of a variety of human malignancies [31]. Consistently, our findings also confirmed that EZH2 was highly expressed in various cancer types, including HCC [32]. Here, we found that high EZH2 levels correlated with the development and progression of HCC. The upregulation of EZH2 predicts poor prognosis in patients with HCC. Moreover, in patients undergoing sorafenib treatment, EZH2 was a factor affecting their survival, indicating that the expression level of EZH2 may distinguish patients who would benefit from sorafenib treatment. Notably, patients with simultaneous high UPK1A-AS1 and EZH2 expression exhibited shorter OS than those with low expression of UPK1A-AS1 and EZH2, suggesting that the combined detection of UPK1A-AS1 and EZH2 expression can better predict the prognosis of patients with HCC.

Piling studies have highlighted that EZH2 participates in cancer cell proliferation by regulating several cell cycle-related genes [33]. GSEA analysis showed that high expression of UPK1A-AS1 was correlated with EZH2 targets that were cell cycle-related, suggesting that UPK1A-AS1 may contribute to HCC progression by regulating EZH2-correlated signaling. Indeed, our current study showed that UPK1A-AS1 plays an oncogenic role by modulating EZH2 activity because the downregulation of EZH2 abrogated the tumor-promoting activity of UPK1A-AS1 in HCC. It has been reported that the subcellular localization of EZH2 correlates with the mechanism of EZH2 oncogenic activity. EZH2 present in the cytoplasm may participate in actin polymerization to influence tumor dissemination [34]. Conversely, EZH2, SUZ12, and EED form a complex in the nucleus and transcriptionally regulate gene expression [35]. The association of EZH2, SUZ12, and EED is responsible for PRC2 activation. Here, we found that UPK1A-AS1 induced the translocation of EZH2 from the cytoplasm to the nucleus. Furthermore, UPK1A-AS1 increased the interaction between EZH2 and SUZ12 that promoted the methylation of lysine 27 in histone H3, indicating that UPK1A-AS1 contributes to the formation and activation of the PRC complex. EZH2-mediated PRC2 activation contributes to the transcriptional silencing of tumor suppressor genes, leading to the activation of NOTCH [36], JAK-STAT [37], or β-catenin signaling pathways [38] and upregulation of cell cycle genes, such as CDK2, CDK4, and CCND1. However, UPK1A-AS1 overexpression did not change the expression of p-STAT3 (data not shown) or β-catenin (Fig. 6g). In contrast, si-EZH2 abolished the upregulation of CDK2, CDK4, and CCND1 caused by UPK1A-AS1, suggesting that UPK1A-AS1 upregulated the aforementioned genes via EZH2, but not EZH2-mediated JAK-STAT and β-catenin signaling activation. Growing evidence has shown that EZH2 can function independently of PRC2 to facilitate transcriptional activation rather than repression [39–41]. EZH2 directly binds to the promoter regions of CCND1 and promotes its transcriptional activation [42]. Whether the UPK1A-AS1-mediated upregulation of CDK2, CDK4, and CCND1 is EZH2-dependent transcriptional activation still requires further investigation.

## Conclusions

Collectively, our findings uncovered the biological function and underlying mechanism of a newly identified lncRNA, UPK1A-AS1, which promotes HCC progression partially dependent on EZH2 by accelerating the cell cycle G1/S transition. Moreover, UPK1A-AS1 was highly expressed in HCC, and the high expression of UPK1A-AS1 predictd poor prognosis in patients with HCC, suggesting that UPK1A-AS1 may serve as a potential biomarker for HCC prognosis and therapy.

## Supplementary Information

**Additional file 1 Figure S1.** UPK1A-AS1 promotes HCC cell proliferation. **Figure S2.** Knocked down of UPK1A-AS1 inhibits G1/S transition of HCC cells. **Figure S3.** Downregulation of EZH2 decreases cell cycle-related genes expression. **Figure S4.** Correlation of EZH2 and its targets in HCC samples. **Figure S5.** UPK1A-AS1 interacts with EZH2. **Figure S6.** UPK1A-AS1 promotes HCC cell proliferation in part by sponging miR-138-5p. **Figure S7.** Expression level of UPK1A-AS1 in normal tissues and cancers. **Figure S8.** Highly expressed EZH2 correlates with poor prognosis in patients with HCC. **Figure S9.** Overexpression of EZH2 predicts poor prognosis in patients with HCC. **Table S1.** Primers and Oligonucleotides used in this study. **Table S2.** Antibodies used in this study.

## Data Availability

The cancer genome atlas program data was downloaded from the National Cancer Institute (https://www.cancer.gov/about-nci/organization/ccg/research/structural genomics/tcga). Gene expression data (GSE10143, GSE14520, GSE22058, GSE54236, GSE64041) were downloaded from Gene Expression Omnibus (http://www.ncbi.nlm.nih.gov/geo).

## Abbreviations

AGO2: Argonaute 2
BSA: Bovine serum albumin
CCK-8: Cell counting Kit
CBTCC: Cell Bank of Type Culture Collection
DAPI: 4, 6-diamidino-2-phenylindole
EZH2: Zeste Homologue 2
ESCC: Esophageal squamous cell carcinoma
FISH: Fluorescence in situ hybridization
GSEA: Gene Sets Enrichment Analysis
GTEx: Genotype-Tissue Expression
GO: Gene Ontology
HCC: Hepatocellular carcinoma
Ig: Immunoglobulin
IF: Immunofluorescence
lncRNA: Long non-coding RNA
LNA: Locked nucleic acid
nt: Nucleotide
OS: Overall survival
PBS: Phosphate-buffered saline
qRT-PCR: RNA extraction and quantitative polymerase chain reaction
RNA-Seq: RNA sequencing
RRC2: Polycomb repressive complex 2
RIP: RNA immunoprecipitation
RISC: RNA-induced silencing complex
siRNA: Small interference RNA
shRNA: Short hairpin RNA
SDS-PAGE: Sodium dodecyl sulfate-polyacrylamide gel
TCGA: The Cancer Genome Atlas
UPK1A-AS1: UPK1A antisense RNA 1
