# Supplementary material for: Long noncoding RNA UPK1A-AS1 indicates poor prognosis of hepatocellular carcinoma and promotes cell proliferation through interaction with EZH2
Source: J Exp Clin Cancer Res. 2020 Oct 29;39:229. doi: 10.1186/s13046-020-01748-y (PMC7596966; doi:10.1186/s13046-020-01748-y)
Supplement: Supplementary file 1 — Additional file 1 Figure S1. UPK1A-AS1 promotes HCC cell proliferation. Figure S2. Knocked down of UPK1A-AS1 inhibits G1/S transition of HCC cells. Figure S3. Downregulation of EZH2 decreases cell cycle-related genes expression. Figure S4. Correlation of EZH2 and its targets in HCC samples. Figure S5. UPK1A-AS1 interacts with EZH2. Figure S6. UPK1A-AS1 promotes HCC cell proliferation in part by sponging miR-138-5p. Figure S7. Expression level of UPK1A-AS1 in normal tissues and cancers. Figure S8. Highly expressed EZH2 correlates with poor prognosis in patients with HCC. Figure S9. Overexpression of EZH2 predicts poor prognosis in patients with HCC. Table S1. Primers and Oligonucleotides used in this study. Table S2. Antibodies used in this study. [file 13046_2020_1748_MOESM1_ESM.docx]

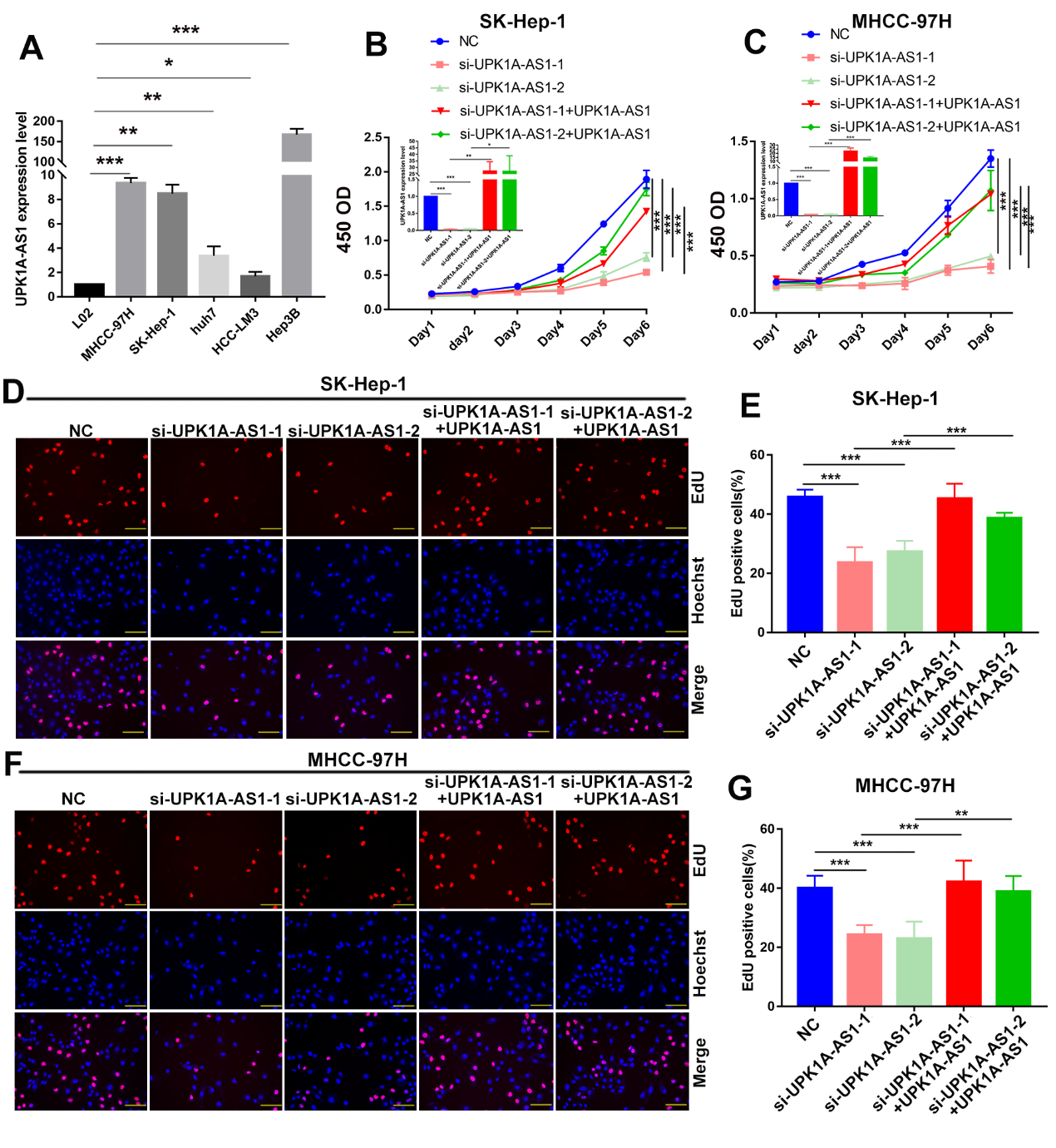


Supplementary Figure 1. UPK1A-AS1 promotes HCC cell proliferation. A. Expression of UP1K-AS1 in human hepatocyte cell line L02 and HCC cell lines MHCC-97H, SK-Hep-1 Huh7, HCC-LM3, and Hep3B was detected by qRT-PCR. B-C. Representative CCK-8 analysis of cell viability after indicated treatments in SK-Hep-1 (B) and MHCC-97H (C) cells (^***^*P* < 0.001). Error bars represent the mean ± S.D. of three independent experiments. D-F. EdU assay showed that overload of UPK1A-AS1 increased ratio of S phase cells reduced by UPK1A-AS1 downregulation in SK-hep-1(D-E) and MHCC-97H (F-G) cells (^**^*P* < 0.01, ^***^*P* < 0.001). Error bars represent the mean ± S.D. of three independent experiments.


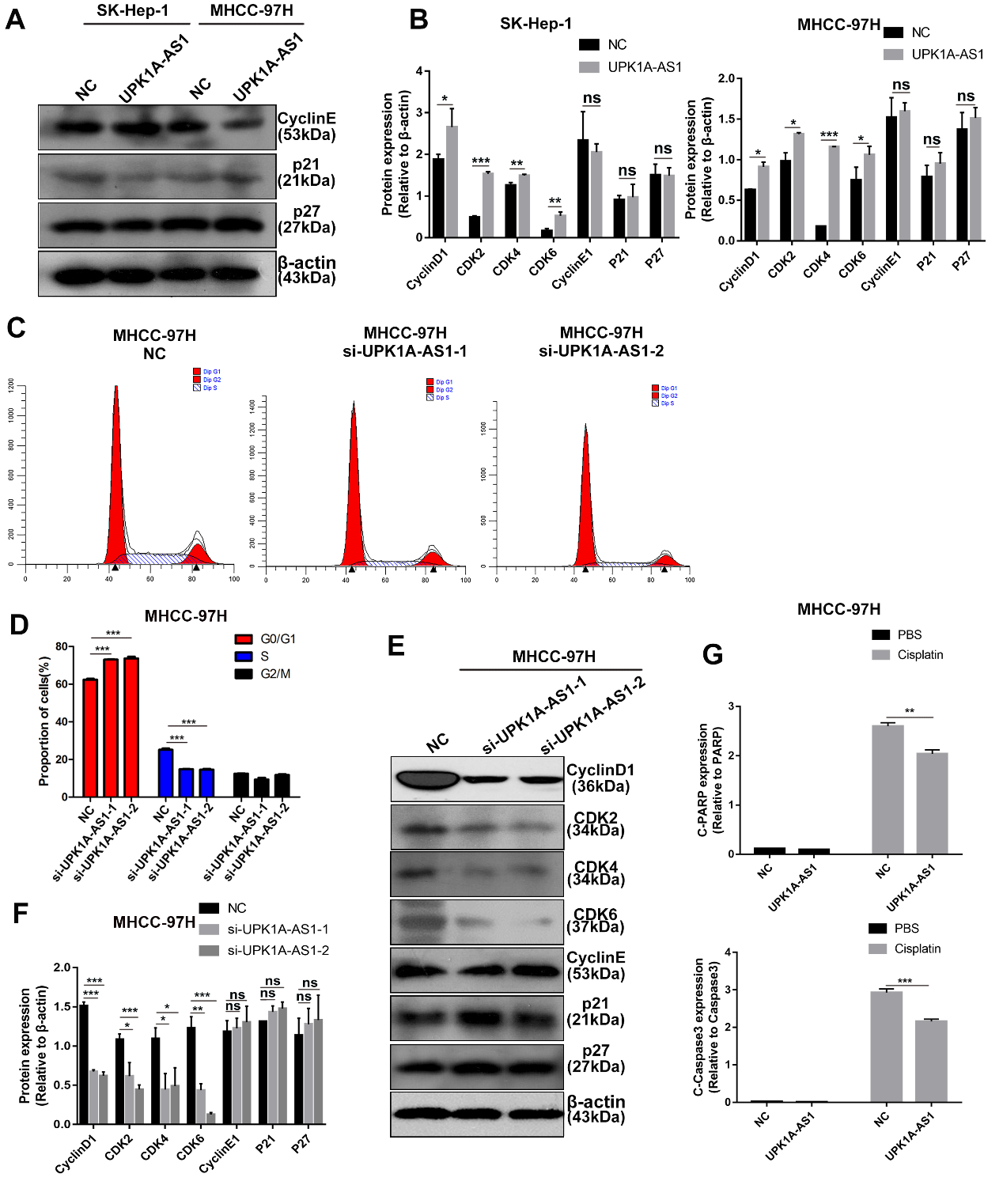


Supplementary Figure 2. Knocked down of UPK1A-AS1 inhibits G1/S transition of HCC cells. A. Expression of the indicated proteins were measured by western blot after overexpression of UPK1A-AS1 in SK-Hep-1 and MHCC-97H cells. B. Statistical analysis of indicated protein expression detected by western blot (^*^*P* < 0.05, ^**^*P* < 0.01, ^***^*P* < 0.001, ns: not significant). Error bars represent the mean ± S.D. of three independent experiments. C-D. Downregulation of UPK1A-AS1 decreased the ratio of cells in S phase compared with negative control in MHCC-97H cells (^***^*P* < 0.001). Error bars represent the mean ± S.D. of three independent experiments. E. Silencing UPK1A-AS1 decreased the expression of CyclinD1, CDK2, CDK4 and CDK6 in MHCC-97H cells. F. Statistical analysis of indicated protein expression detected by western blot (^*^*P* < 0.05, ^**^*P* < 0.01, ^***^*P* < 0.001, ns: not significant). Error bars represent the mean ± S.D. of three independent experiments. G. Fold change of indicated protein expression in MHCC-97H cells with or without cisplatin treatment detected by western blot (^**^*P* < 0.01, ^***^*P* < 0.001). Error bars represent the mean ± S.D. of three independent experiments.


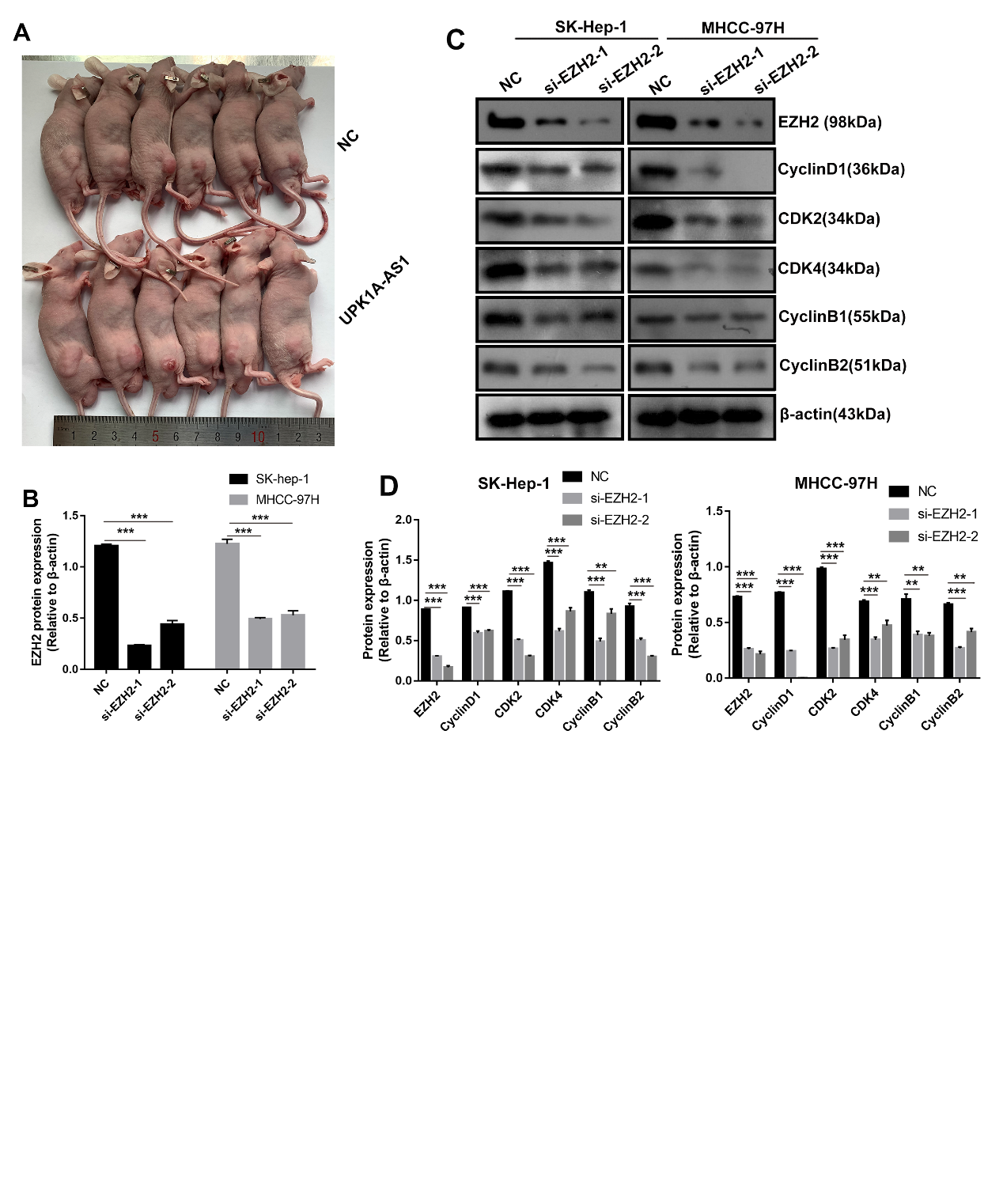


Supplementary Figure 3. Downregulation of EZH2 decreases cell cycle-related genes expression. A. Morphologies of tumor showing overexpression of UPK1A-AS1 promoted tumor growth in HCC. B. Knockdown efficacy of EZH2 by siRNAs detected by western blot. (^***^*P* < 0.001). Error bars represent the mean ± S.D. of three independent experiments. C-D. Expression of the indicated proteins were detected in SK-Hep-1 and MHCC-97H cells with EZH2 downregulation using western blot assay. (^*^*P* < 0.05, ^**^*P* < 0.01, ^***^*P* < 0.001). Error bars represent the mean ± S.D. of three independent experiments.


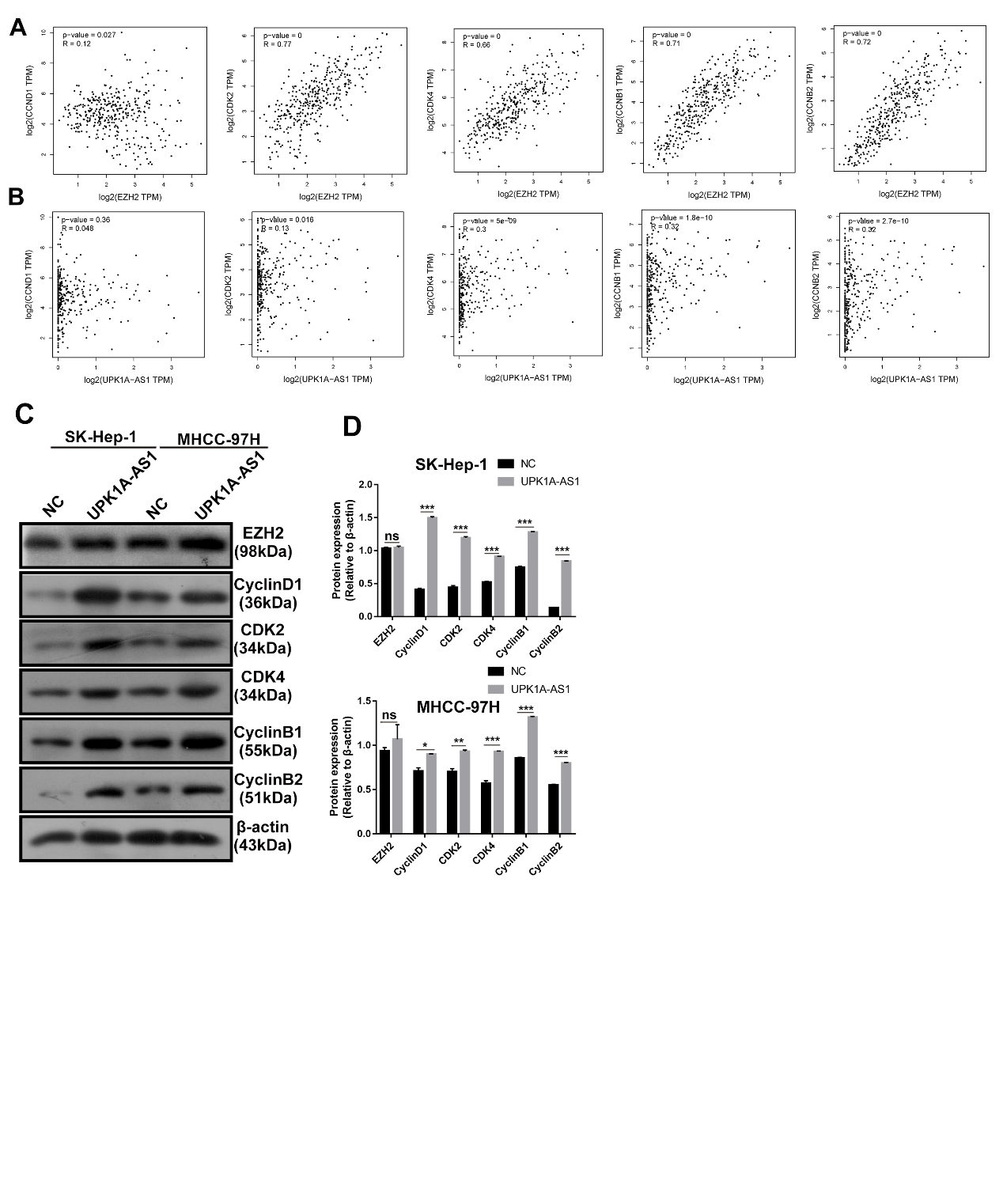


Supplementary Figure 4. Correlation of EZH2 and its targets in HCC samples. A. Correlation of EZH2 and its targets in HCC samples from TCGA dataset. B. Correlation of UPK1A-AS1 and targets of EZH2 that was cell cycle related in HCC samples from TCGA dataset. C-D. Expression of the indicated proteins were detected in SK-Hep-1 and MHCC-97H cells with UPK1A-AS1 overexpression using western blot assay (^*^*P* < 0.05, ^**^*P* < 0.01, ^***^*P* < 0.001). Error bars represent the mean ± S.D. of three independent experiments.


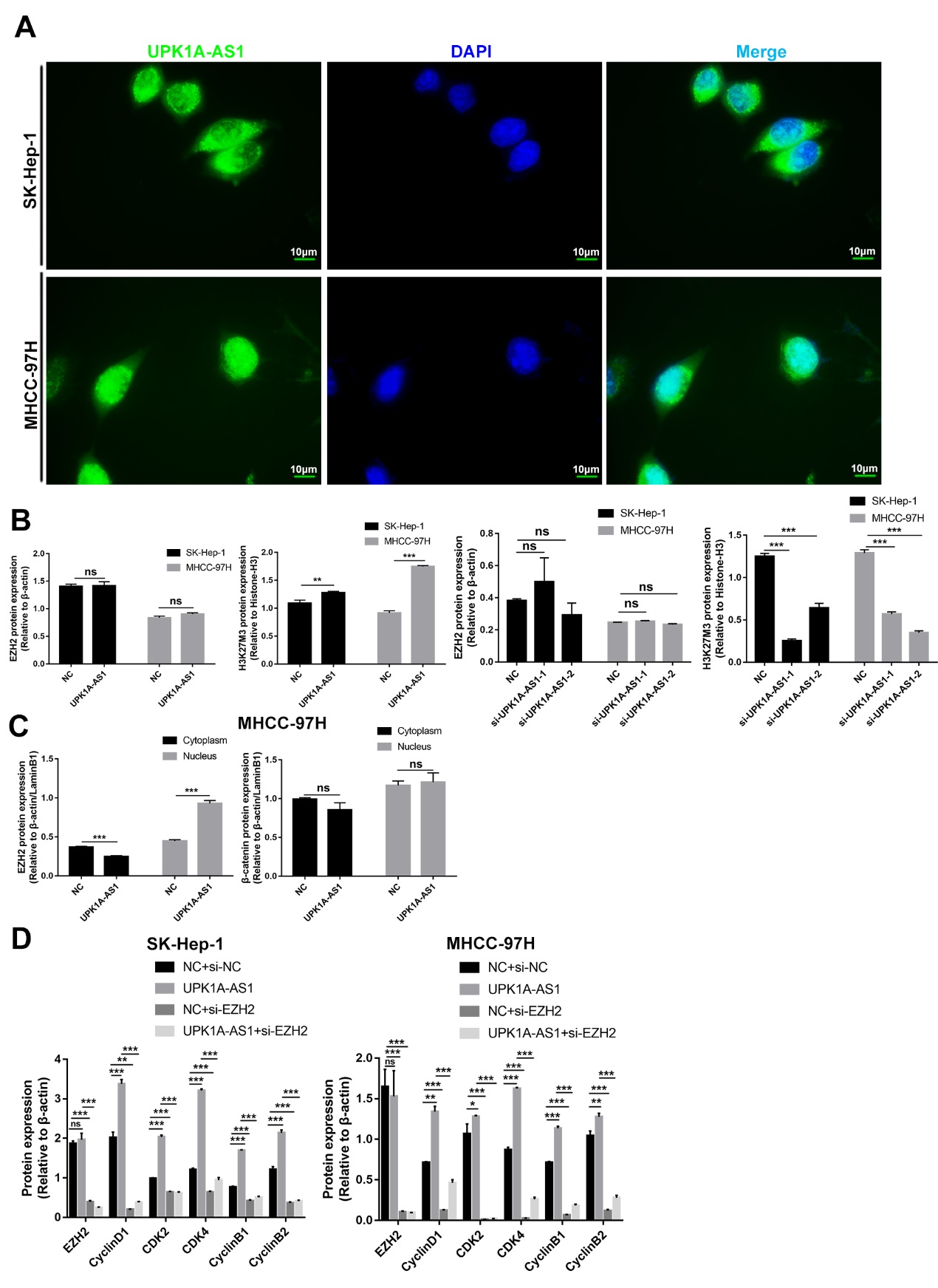


Supplementary Figure 5. UPK1A-AS1 interacts with EZH2. A. UPK1A-AS1 was distributed in both nucleus and cytoplasm analyzed by FISH assay. UPK1A-AS1 was labeled by FITC (green), nucleus was stained by DAPI (blue). B-D. Statistical analysis of indicated protein expression detected by western blot (^*^*P* < 0.05, ^**^*P* < 0.01, ^***^*P* < 0.001, ns: not significant). Error bars represent the mean ± S.D. of three independent experiments.


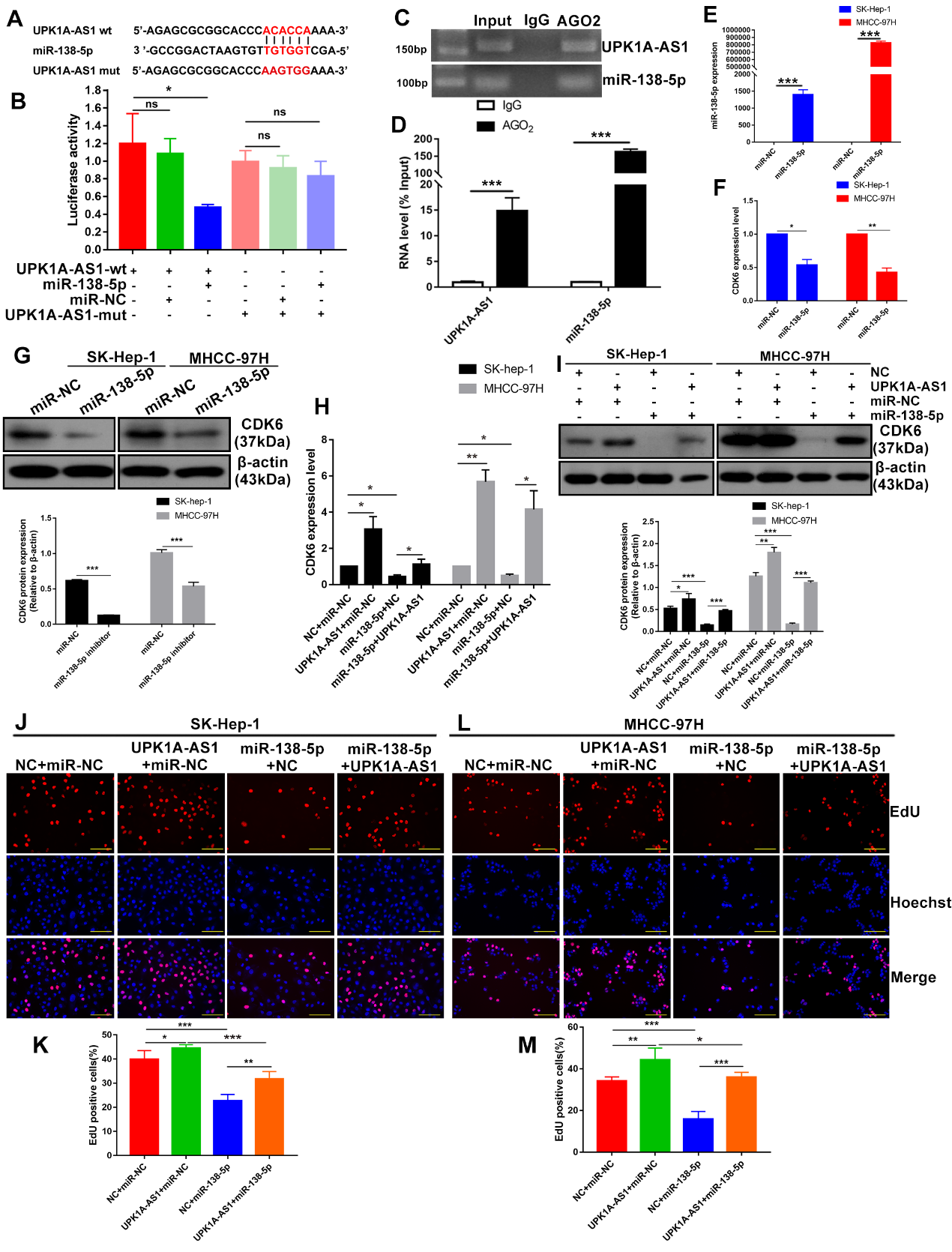


Supplementary Figure 6. UPK1A-AS1 promotes HCC cell proliferation in part by sponging miR-138-5p. A. Schema representing the functional interaction between UPK1A-AS1 and miR-138-5p as predicted by miRcode. B. Wild type and mutant UPK1A-AS1 sequences were cloned and inserted into psiCHEK-2.0 vectors and co-transfected with miR-138-5p into MHCC-97H cells. C-D. RNA immunoprecipitation with an anti-AGO2 antibody was used to determine endogenous AGO2 binding to miR-138-5p and UPK1A-AS1 in MHCC-97H cells. IgG was used as control. The level of UPK1A-AS1 and miR-138-5p were detected by qRT-PCR (^***^*P* < 0.001). Error bars represent the mean ± S.D. of three independent experiments. E. miR-138-5p was overexpressed in SK-Hep-1 and MHCC-97H cells transfected with miR-138-5p mimics. (^***^*P* < 0.001). Error bars represent the mean ± S.D. F-G. The mRNA (F) and protein (G) expression of CDK6 was measured in SK-Hep-1 and MHCC-97H cells transfected with miR-138-5p mimics. (^*^*P* < 0.05, ^**^*P* < 0.01, ^***^*P* < 0.001). Error bars represent the mean ± S.D. of three independent experiments. H-I. The mRNA (H) and protein (I) expression of CDK6 was measured in SK-Hep-1 and MHCC-97H cells with indicted treatments. (^*^*P* < 0.05, ^**^*P* < 0.01, ^***^*P* < 0.001). Error bars represent the mean ± S.D. of three independent experiments. J-M. EdU assay showed that overexpression of miR-138-5p decreased ratio of S phase cells increased by UPK1A-AS1 overexpression in SK-hep-1(J-K) and MHCC-97H (L-M) cells (^*^*P* < 0.05, ^**^*P* < 0.01, ^***^*P* < 0.001). Error bars represent the mean ± S.D. of three independent experiments.


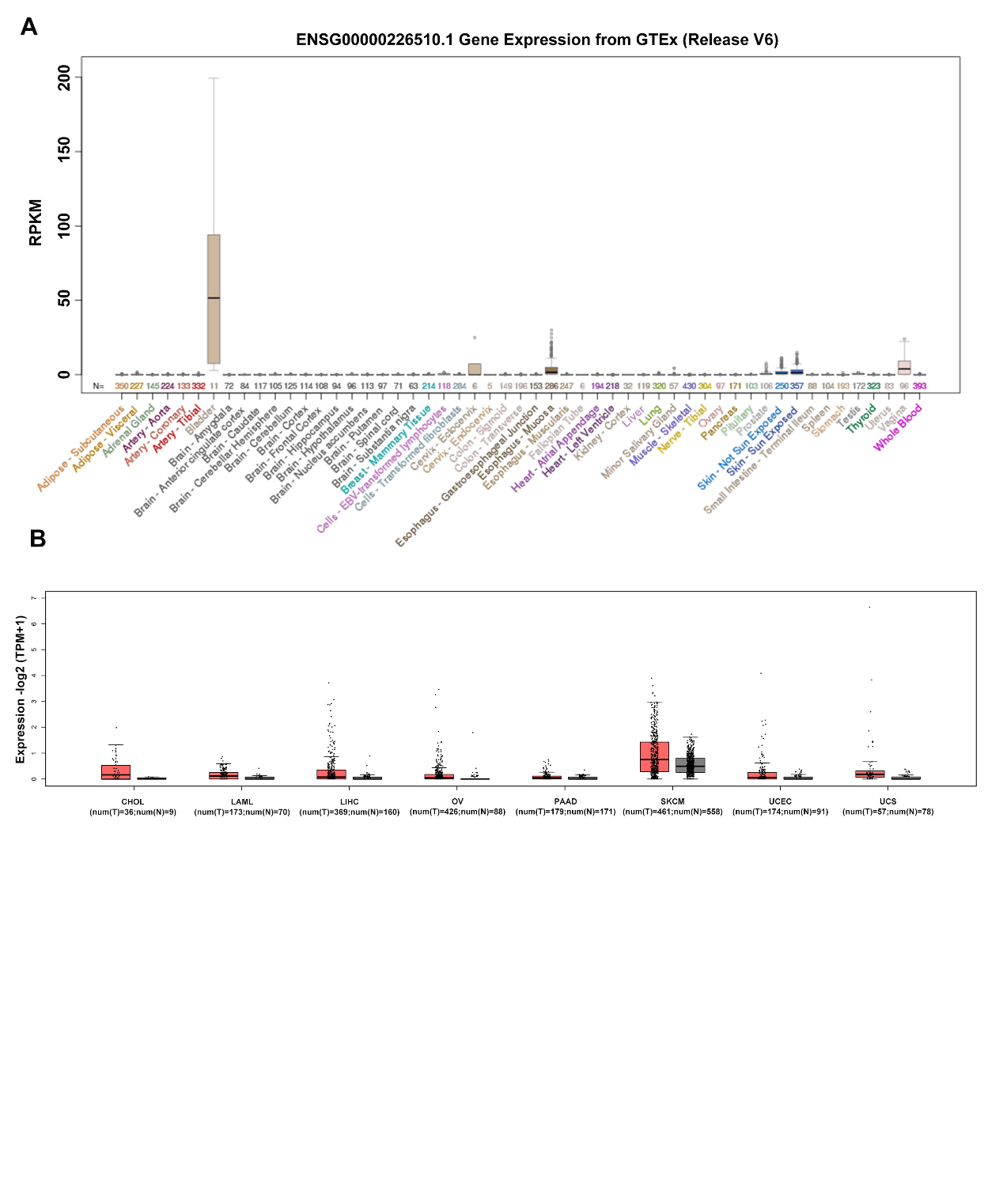


Supplementary Figure 7. Expression level of UPK1A-AS1 in normal tissues and cancers. A. Expression level of UPK1A-AS1 in normal tissues from RNA-seq of GTEx. B. RNA-seq from TCGA datasets showed that UPK1A-AS1 was upregulated in cancers.


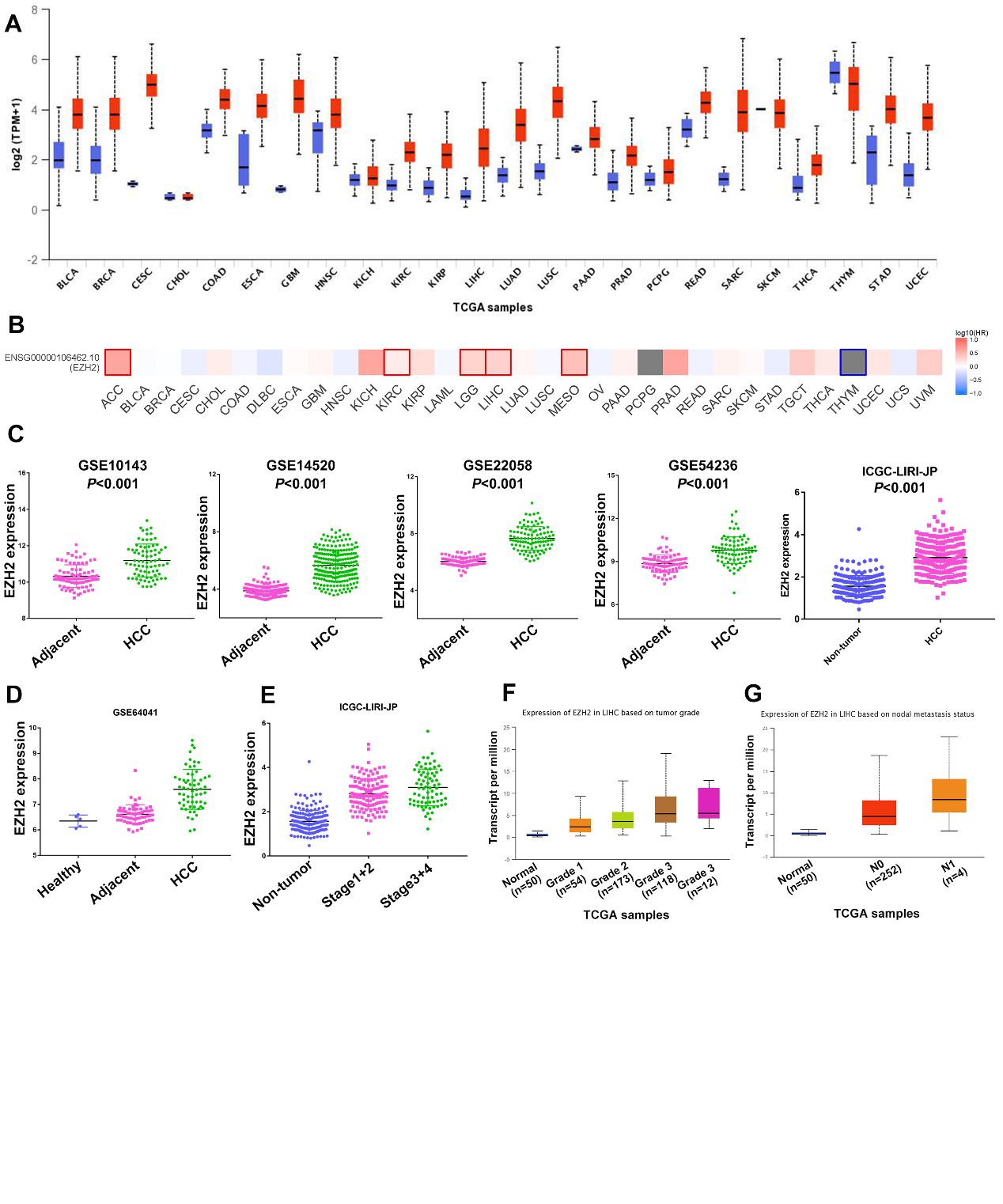


Supplementary Figure 8. Highly expressed EZH2 correlates with poor prognosis in patients with HCC. A. EZH2 was upregulated in various cancer analyzed from RNA-seq of TCGA. B. Overexpression of EZH2 correlated with poor prognosis in various cancers. C. The expression of EZH2 in HCC tissues and non-tumors from a series of HCC datasets (*P* < 0.001). D. Expression of EZH2 in Healthy liver, adjacent non-tumor tissues and HCC tissues. E. Expression of EZH2 in non-tumor tissues and HCC tissues of different stage. F. Expression of EZH2 in HCC with different histologic grade. G. Expression of EZH2 in HCC with or without nodal metastasis status.


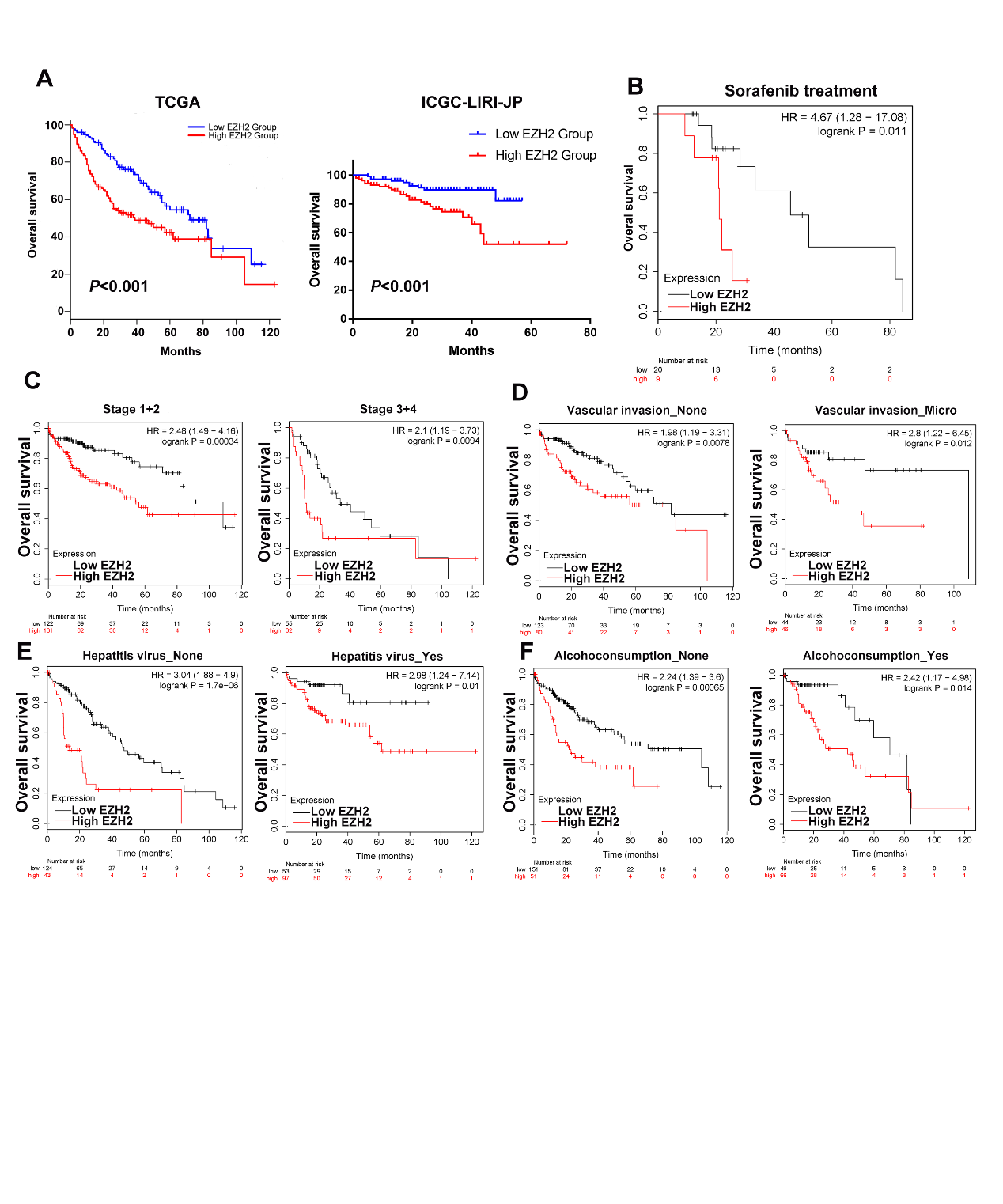


Supplementary Figure 9. Overexpression of EZH2 predicts poor prognosis in patients with HCC. A. Kaplan-Meier analysis of overall survival in the TCGA and ICGC-LIRI-JP dataset on the basis of EZH2 expression (*P* < 0.05). B-F. Stratified analysis showed the correlation of EZH2 expression and overall survival in indicated groups in HCC from TCGA dataset from Kaplan Meier-plotter website (http://kmplot.com/analysis).

**Table S1 Primers and Oligonucleotides used in this study**

| UPK1A-AS1 | F:5’-AGAGCGGTGGGTTAGGAA-3’  R:5’-GGGCAGATGGACCAAGCA-3’ |
| --- | --- |
| β-actin | F:5’-TCAAGATCATTGCTCCTCCTGA-3’  R:5’-CTCGTCATACTCCTGCTTGCTG-3’ |
| CCND1 | F:5’-AGCTGTGCATCTACACCGAC-3’  R:5’-AATGAACTTCACATCTGTGGCA-3’ |
| CDK2 | F:5’-TTGCTGAGATGGTGACTCG-3’  R:5’-TGTTAGGGTCGTAGTGCAGC-3’ |
| CDK4 | F:5’- AGCTTGCGGCCTGTGTCTAT -3’  R:5’- ATCAAGGGAGACCCTCACGC-3’ |
| CDK6 | F:5’-CCTGCAGGGAAAGAAAAGTGCAA-3’  R:5’-CCTCGAAGCGAAGTCCTCAA-3’ |
| CCNE1 | F:5’-CCCATCATGCCGAGGGAG-3’  R:5’-TATTGTCCCAAGGCTGGCTC-3’ |
| CCNB1 | F:5’-GCACTTCCTTCGGAGAGCAT-3’  R:5’-TGTAGAGTTGGTGTCCATTCAC-3’ |
| CCNB2 | F:5’- TGTCCTCCCTTTTCAGTCCG -3’  R:5’- CCAAATCACTGGACACCGTC-3’ |
| si-UPK1A-AS1-1 | 5’-GGAGATGCTTGGTCCATCT-3’ |
| si-UPK1A-AS1-2 | 5’-CCTTGACAGTGCTGTTATT-3’ |
| Sh-UPK1A-AS1-2 | 5’-GTGAGCAGAGGCCAGGAGAGA-3’ |
| Sh-UPK1A-AS1--3 | 5’-ATGGCCATAAACATTACAAAT-3’ |

**Table S2 Antibodies used in this study**

| Antibodies Source Purpose Identifier |
| --- |
| Mouse Monoclonal anti-CyclinD1 Santa Cruz WB Cat#sc-8396  Rabbit Polyclonal anti-CDK2 Proteintech WB Cat#10122-1-AP  Rabbit Polyclonal anti-CDK4 Proteintech WB Cat#11026-1-AP  Rabbit Polyclonal anti-CDK6 Abcam WB Cat#ab124821  Rabbit Monoclonal anti-CyclinE1 Santa Cruz WB Cat#sc-377100  Rabbit Polyclonal anti-p21 Proteintech WB Cat#10355-1-AP  Rabbit Polyclonal anti-p27 Proteintech WB Cat#25614-1-AP  Rabbit Polyclonal anti-CyclinB1 Proteintech WB Cat#55004-1-AP  Rabbit Polyclonal anti-CyclinB2 Proteintech WB Cat#21644-1-AP  Rabbit Polyclonal anti-PARP Abcam WB Cat#ab32138  Rabbit Polyclonal anti-caspase3 Abcam IF Cat#ab13847  Rabbit Polyclonal anti-EZH2 CST WB/IP/IF Cat#5246  Rabbit Polyclonal anti-H3K27M3 ABclonal WB Cat#A2363  Mouse Monoclonal anti-β-actin Santa Cruz WB Cat#sc-47778  Rabbit Polyclonal anti-Histone-H3 Proteintech WB Cat#17168-1-AP  Rabbit Polyclonal anti-β-catenin Proteintech WB Cat#17565-1-AP  Rabbit Polyclonal anti-LaminB1 Proteintech WB Cat#12987-1-AP  Rabbit Polyclonal anti-SUZ12 ABclonal WB Cat#A7786 |
|  |
